# Inhibition of lysosomal phospholipase A2 predicts drug-induced phospholipidosis

**DOI:** 10.1016/j.jlr.2021.100089

**Published:** 2021-06-01

**Authors:** Vania Hinkovska-Galcheva, Taylour Treadwell, Jonathan M. Shillingford, Angela Lee, Akira Abe, John J.G. Tesmer, James A. Shayman

**Affiliations:** 1 Department of Internal Medicine, University of Michigan Medical School, University of Michigan, Ann Arbor, MI, USA; 2 Departments of Biological Sciences and Medicinal Chemistry and Pharmacology, Purdue University, West Lafayette, IN, USA

**Keywords:** Acyltransferase, 1-*O*-acylceramide, lysosome, phospholipase A2 group XV, drug-induced phospholipidosis, drug toxicity, cationic amphiphilic drugs, drug development, high-throughput screening, amiodarone, CAD, cationic amphiphilic drug, DIP, drug-induced phospholipidosis, DODPC, 1,2-di-*O*-octadecenyl-*sn*-glycero-3-phosphocholine, DOPC, 1,2-dioleoyl-palmitoyl-*sn*-glycero-3-phosphocholine, HPTLC, high-performance thin layer chromatography, LPLA_2_, lysosomal phospholipase A2, NAS, *N*-acetyl-sphingosine, pNPB, *p*-nitro-phenyl butyrate

## Abstract

Phospholipidosis, the excessive accumulation of phospholipids within lysosomes, is a pathological response observed following exposure to many drugs across multiple therapeutic groups. A clear mechanistic understanding of the causes and implications of this form of drug toxicity has remained elusive. We previously reported the discovery and characterization of a lysosome-specific phospholipase A2 (PLA2G15) and later reported that amiodarone, a known cause of drug-induced phospholipidosis, inhibits this enzyme. Here, we assayed a library of 163 drugs for inhibition of PLA2G15 to determine whether this phospholipase was the cellular target for therapeutics other than amiodarone that cause phospholipidosis. We observed that 144 compounds inhibited PLA2G15 activity. Thirty-six compounds not previously reported to cause phospholipidosis inhibited PLA2G15 with IC_50_ values less than 1 mM and were confirmed to cause phospholipidosis in an in vitro assay. Within this group, fosinopril was the most potent inhibitor (IC_50_ 0.18 μM). Additional characterization of the inhibition of PLA2G15 by fosinopril was consistent with interference of PLA2G15 binding to liposomes. PLA2G15 inhibition was more accurate in predicting phospholipidosis compared with in silico models based on pKa and ClogP, measures of protonation, and transport-independent distribution in the lysosome, respectively. In summary, PLA2G15 is a primary target for cationic amphiphilic drugs that cause phospholipidosis, and PLA2G15 inhibition by cationic amphiphilic compounds provides a potentially robust screening platform for potential toxicity during drug development.

Phospholipidosis is the excess storage of phospholipids within lysosomes. Drug-induced phospholipidosis (DIP), in distinction to inherited forms of lysosomal phospholipid accumulation such as those associated with disorders such as Niemann–Pick C disease, represents an acquired lysosomal disorder (1, 2). DIP most often involves the lung, liver, or kidney where it is associated with pulmonary fibrosis, hepatic steatosis or steatohepatitis, and acute or chronic kidney injury, respectively. Phospholipidosis often, but not always, results from exposure to basic cationic amphiphilic drugs (CADs). DIP is measured experimentally by use of in vitro or in vivo assays and is often observed in clinical settings. It is among the most common forms of drug toxicity as it is associated with exposure to more than 50 FDA-approved agents. When DIP is detected in preclinical screening studies, an otherwise promising compound may be abandoned. If DIP is found in patients under treatment with a specific drug, then the therapeutic is often discontinued. Research on DIP has been dominated by three overarching questions. First, what are the mechanisms responsible for DIP? Second, what chemical properties of a candidate compound can be used to predict phospholipidosis and used as a guide for further development? Third, what significant short- and long-term toxicities are the specific consequences of DIP?

With regard to the first question, several mechanisms have been proposed as the basis DIP. These include the stimulation of phospholipid synthesis (3), the direct binding of CADs to lysosomal phospholipases with inhibition of these enzymes by competitive or allosteric mechanisms (4), the inhibition of lysosomal trafficking to lysosomes (5), the displacement of phospholipases from the lysosomal membrane with secondary degradation by lysosomal proteases (6), and the binding of CADs to phospholipids with prevention of their degradation (7). Lysosomal phospholipase A1, A2, and C activities have been previously associated with DIP. However, to date only three phospholipases are known to be lysosome-based. They include acid sphingomyelinase (8), phospholipase D3 (9), and lysosomal phospholipase A2 (10).

With regard to the second question, efforts to predict DIP have generally followed two strategies. The first approach has employed analyses in which the physical properties of drugs are correlated with empirically observed phospholipidosis (11, 12, 13, 14). The second strategy has used the development of novel in vitro assays that can be applied to the screening of individual drug candidates or chemical libraries to predict phospholipidosis potential. These assays include those that detect lipid accumulation in cell lines or that specifically measure lysosome associated lipid biomarkers such as bis(monoacylglycerol)phosphate (15) or gene expression profiling (16). The assessment of these various in silico and in vitro strategies is limited by the absence of proof of a mechanism responsible for DIP.

With regard to the third question, a determination of the pathological significance of phospholipidosis has been limited by the lack of identification and characterization of a specific target or targets of compounds that cause DIP. Identifying the cellular target or targets responsible for DIP as distinguished from toxicities resulting from separate off-target effects would represent a significant step in understanding and managing this form of drug toxicity.

Our group identified an enzyme with 1-O-acyl-ceramide synthase activity and subsequently characterized a purified enzyme as lysosomal phospholipase A2 (LPLA_2_), now designated PLA2G15 (17, 18, 19). LPLA_2_ has an acidic pH optimum and colocalizes with lysosomes and late endosomes. Loss of function of LPLA_2_ in mice results in alveolar macrophage foam cell formation and surfactant accumulation, a phenotype similar to that observed with amiodarone-associated phospholipidosis (20). In subsequent work we reported that amiodarone is a potent inhibitor of LPLA_2_, but does so by inhibition of electrostatic charge interactions between the hydrolase and anionic phospholipids (21). This mechanism of action was further substantiated by our determination of the crystal structure of LPLA_2_ and the identification of critical residues in the lipid membrane-binding domain (22).

Based on these studies we considered whether the inhibition of LPLA_2_ by cationic amphiphilic compounds is a more general mechanism for DIP. We assayed two libraries of small molecules for their ability to inhibit LPLA_2_ and correlate this inhibition with physical properties of these compounds used by others as the basis for predictive models of phospholipidosis. The first library consisted of drugs known to cause phospholipidosis in either in vitro or in vivo studies based on published reports. The second library consisted of compounds for which DIP has not been previously reported. We observed that inhibition of LPLA_2_ strongly correlates with drugs reported to cause phospholipidosis and have identified drugs that have not previously known to cause phospholipidosis, not all of which are cationic amphiphiles.

## Materials and Methods

### Materials

1,2-dioleoyl-palmitoyl-*sn*-glycero-3-phosphocholine (DOPC), 1,2-di-*O*-octadecenyl-*sn*-glycero-3-phosphocholine (DODPC), and brain porcine sulfatide ammonium salt were purchased from Avanti Polar Lipids (Birmingham, AL). *p*-Nitro-phenyl butyrate (pNPB) was from Sigma (St. Louis, MO). Purified recombinant mouse LPLA_2_ was produced by Proteos Inc. (Kalamazoo, MI) as previously reported (22). High-performance thin layer chromatography (HPTLC) silica gel plates (10 × 20 cm) were from Merck KG^@^A (Darmstadt, Germany). All cationic amphiphilic drugs and controls used in this study were obtained from Sigma-Aldrich (St. Louis, MO) with the following exceptions ay-9944 and suramin from Calbiochem (San Diego, CA), and clenbuterol and yohimbine were from Cayman Chemicals (Ann Arbor, MI).

### Transacylase activity of LPLA_2_

The LPLA_2_ activity assay is based on the following principles (23). LPLA_2_ is uniquely characterized as having an acidic pH optimum and as a transacylase recognizing short-chain lipophilic alcohols as acceptors. Based on these properties, short-chain 1-O-acyl-ceramides are unique products of this reaction. Because LPLA_2_ binds preferentially to negatively charged liposomes, sulfatide was included in the liposomes but is not itself a substrate and does not function as a cofactor for lysosomal hydrolases. The transacylase reaction is based on the unique property of LPLA_2_ to transfer an acyl group from the *sn*-2 or *sn*-1 position of a glycerophospholipid to *N*-acetyl-sphingosine (NAS) forming 1-*O*-acyl-N-acetylsphingoine (1-*O*-acyl-NAS) (18, 22, 24). 1-*O*-acyl-NAS is not known to be a product of any other enzyme. The reaction mixture included 50 mM sodium citrate buffer (pH 4.5), 10 μg/ml bovine serum albumin, and liposomes consisting of 38 μM N-acetyl-sphingosine, 127 μM DOPC, 12.7 μM sulfatide, and test compound in a total volume of 0.5 ml. The test compounds were dissolved in DMSO. The final DMSO concentration in the reaction mixture was 0.125%. The reaction was initiated by the addition of recombinant LPLA_2_ protein (30 ng) and carried out at 37 ˚C for 10 min. The reaction was terminated by the addition of 3 ml chloroform/methanol (2/1, v/v), followed by 0.3 ml of 9% (w/v) NaCl. After centrifugation for 7 min at 1800 × g, the resulting lower layer was transferred to new tube and dried under stream of nitrogen gas. The dried lipid was dissolved in 40 μl of chloroform/methanol (2/1, v/v) and applied to HPTLC plates. HPTLC plates were run in chloroform/acetic acid (9/1, v/v). The plates were dried and soaked in 8% (w/v) CuSO_4_.5H_2_O, 6.8% (v/v) H_3_PO_4_, and 32% (v/v) methanol and then charred for 15 min in an oven at 150 ˚C. Scanned plates were analyzed by NIH ImageJ 1.651j8 (National Institutes of Health).

### LPLA_2_ esterase assay

pNPB was used to directly measure the activity of LPLA_2_. pNPB is a water-soluble substrate that can directly access the catalytic site in the absence of liposomes (25). A reaction mixture of pNPB (0.2 mM) and cationic amphiphilic compounds at varying concentrations in sodium citrate buffer (pH 4.5) was prepared and prewarmed to 37°C for 5 min in a total volume of 500 μl. The reaction was initiated by the addition of recombinant LPLA_2_ (5 μg). At predetermined times, 120 μl of the reaction mixture was transferred to a tube containing 120 μl of 0.2 M NaHCO_3_ and kept on ice. The cold reaction product was subsequently warmed to 37°C, and the absorbance of the reaction product, *p*-nitrophenoxide, was measured at 400 nm with a Beckman Du-640 spectrophotometer.

### Liposome LPLA_2_ cosedimentation assay

Liposomes consisting of DOPC and sulfatide (10:1 M ratio, 127 μM total lipid) were incubated with 5 μg of LPLA_2_ in 500 μl 50 mM sodium citrate, at pH 4.5 for 30 min on ice. The reaction mixture was then centrifuged for 1 h at 150,000 *g* at 4°C. The resulting precipitate was rinsed with cold 50 mM sodium citrate pH 4.5 and dissolved with 40 μl of SDS-PAGE sample buffer. The sample was separated by using 10% SDS-PAGE. After electrophoresis, LPLA_2_ was detected with Coomassie brilliant blue. Band quantification was performed with *ImageJ* software I1.651j8 (25).

### LPLA_2_ thermal stability measurement

A thermal stability assay was employed to determine the melting point (T_m_) of LPLA_2_ (26). An incubation mixture consisting of 2.5 μl of 8x SYPRO Orange, 1 μg of LPLA_2_ in 50 mM Na citrate at pH 4.5, and ddH_2_O in a final volume of 20 μl was added to wells of a 48-well thin-wall PCR plate. The plates were sealed with Optical-Quality Sealing Tape (Bio-Rad) and heated in a Real-Time PCR Detection System Life Technology (Thermo Fisher, Ann Arbor, MI) from 20 to 90°C in steps of 0.2°C. T_m_ values were calculated as the inflection point of the melting curve using the instrument software.

### Screening phospholipidosis assay

The assay was modified from one reported previously (27). MDCK cells were seeded in 100 μl culture medium at cell density 3,000 cells per well in 96-well black-walled clear bottom Greiner micro plates (Sigma-Aldrich) and were allowed to adhere overnight. Cell culture medium was replaced with phospholipidosis staining solution (1:1,000 dilution) of LipidTOX Red Phospholipidosis detection reagent (Invitrogen), and simultaneously with different concentrations of fosinopril or amiodarone in total volume of 100 μl. Compounds were prepared as stock solutions at 200-fold higher concentration than the desired top concentration (solvent concentration maintained at 0.5%). Compound treatment was performed for 24 h with 5% CO_2_ at 37°C. Then the culture medium was removed and cells were fixed with 100 μl, fixation solution consisting of 4% formaldehyde in phosphate buffered saline (PBS). After washing, cells were incubated with 1 drop of NucBlue Live (NBL) for 20 min. Cells were washed three times with PBS, and fluorescence image acquisition was performed using the Molecular Devices (San Jose, CA) spectrophotometer. Cell nuclei fluorescence was detected using a 410–480 nm emission filter, red phospholipidosis detection was performed using 549–615 nm emission filter.

### Image acquisition and processing

Ninety six-well plates were visualized under a Leica DM IRB microscope and images acquired with an Olympus DP70 camera via Olympus DP Manager software. All images were identically adjusted in GNU Image Manipulation Program to improve background and overall image clarity postacquisition.

### LipidTOX red particle quantification

Images were quantified utilizing ImageJ as follows. Images were initially processed with the Subtract Background feature with a rolling ball radius of 50 pixels. Following conversion to 8 bit, images were subjected to Auto Local Threshold processing using the Bernsen algorithm with a radius of 15. Particles were subsequently quantified and analyzed utilizing the Analyze Particle feature. A total of six 10x fields (2 per triplicate) were quantified with an average of over 4,000 cells per field.

### Statistical analysis

Data from at least three independent experiments were analyzed with a paired *t* test in GraphPad Prism 7 and expressed as mean ± SD. The differences between control and treated samples were considered statistically significant at *P* < 0.05.

## Results

A library of 163 compounds was assembled and assayed for inhibition of LPLA_2_. One hundred and nine compounds were identified via literature review as causing phospholipidosis based on either in vitro or in vivo assays (Table 1). In the latter case, the animal species employed is indicated. These compounds were chosen represent a wide spectrum of therapeutic indications, having a range of pKa and ClogP that fell within and outside of values commonly associated with DIP and in which the lysosomal pathology is observed across a range of organs. Most, but not all, of the compounds are cationic amphiphiles, and several are central nervous system penetrant. A second set of 54 compounds was assayed representing drugs for which no reports of phospholipidosis were found but which were representative of a similar spectrum of chemical properties (Table 2). Included in this set were metabolites chosen as negative controls (glucose, leucine, and uridine). The primary clinical indications listed in these tables are consistent with a wide range of cellular targets for these compounds.

**Table 1:** Test compounds reported to cause phospholipidosis

Generic name	UPAC Designation	CAS Number	Indication	ClogP	pKa (basic)	Ploemen Value	Pred Ploemen	Pred modified Ploemen	LPLA2 IC50 (μM)	In Vitro PLD	In Vivo PLD	Refs
Alprenolol	1-(o-allylphenoxy)-3-(isopropylamino)-2-propanol	13707-88-5	Antihypertensive, antiarrhythmic, sympatholytic agent	3.1	9.67	103	+	+	172.7	Yes		(28, 29)
Alverine	ethyl bis (3-phenylpropyl)amine	150-59-4	Antidiarrheal	5.73	10.44	142	+	+	40.01	Yes		(28, 30)
Ambroxol	2-amino-3,5-dibromo-*N*-(*trans*-4-hydroxycyclohexyl) benzylamine	28828-92-4	Mucolytic	3.72	9.01	95	+	+	59	Yes		(28, 31)
Amiodarone	{2-[4-(2-butyl-1-benzofuran-3-carbonyl)-2,6-diiodophenoxy] ethyl} diethyl amine	1951-25-3	Antiarrhythmic	7.57	8.47	130	+	+	8.3	Yes	H,R	(16, 25, 27, 28, 32, 33, 34, 35, 36, 37, 38)
Amitriptyline	dimethyl(3-{tricyclo[9.4.0.0^3^,^8^]pentadeca-1(15),3,5,7,11,13-hexaen-2-ylidene}propyl)amine	50-48-6	Antidepressant	5.1	9.76	121	+	+	14.7	Yes	R	(11, 16, 27, 28, 35, 36, 39, 40)
Amorolfine	2R,6S-2,6-dimethyl-4-(2-{[4-(2-methylbutan-2-yl) phenyl] methyl}propyl)morpholine	78613-35-1	Antifungal	5.62	8.49	104	+	+	41.47	Yes		(28, 30)
Anastrozole	2;2″-[5-(1H-1;2;4-Triazol-1-ylmethyl)-1,3-phenylene]bis(2-methyl-propiononitrile)	120511-73-1	Chemotherapy	2.31	2	9.3	-	-	4.82	Yes		(41, 42)
Astemizole	1-(4-fluorobenzyl)-2-(1-[4-methoxyphenethyl]piperidin-4-yl)aminobenzimidazole	68844-77-9	Antihistamine	5.92	8.75	112	+	+	8.19	Yes		(28, 33, 40)
ay-9944	*trans*-1,4- *bis*(2-chlorobenzylaminomethyl) cyclohexane	366-93-8	Hypocholestrol-emic	6.4	9.1	124	-	+	116	Yes	R,Ra, M	(16, 43, 44, 45, 46)
Benzbromarone	2,6-dibromo-4-(2-ethyl-1-benzofuran-3-carbonyl)phenol	3562-84-3	Xanthine oxidase inhibitor	5.52	-3.8	30.5	-	-	3.8	Yes		(28, 32)
Benfluorex	*N*-(1-methyl-2-(3-[trifluoromethyl]-phenyl)ethyl)amino ethanol benzoate ester	23642-66-2	Anorectic and hypolipidemic	4.26	9.14	102	+	+	19.9	Yes		(35, 47, 48)
Bepridil hydrochloride	1-isobutoxy-2-pyrrolidino-3-(N-benzylanilino) propane hydrochloride	74764-40-2	Calcium channel blocker	5.33	9.16	112	+	+	7.17	Yes		(28, 36)
Betaxolol	1-{4-[2-(cyclopropylmethoxy) ethyl] phenoxy}-3-[(propan-2-yl) amino]propan-2-ol	63659-18-7	Beta blocker	2.81	9.67	101	+	+	0	Yes		(28, 40)
Bromhexine	2-amino-3,5-dibromo-*N*-cyclohexyl-*N*-methylbenzylamine hydrochloride	611-75-6	Mucolytic	4.08	9.32	104	+	+	30.78	Yes		(28, 31, 49, 50)
Buclizine	1-((4-chlorophenyl)phenylmethyl)-4-((4-(1,1-dimethylethyl)phenyl)methyl)piperazine	82-95-1	Antihistamine	6.16	8.04	103	+	+	9.13	Yes		(12, 28)
Bromocriptine	(4R,7R)-10-bromo-N-[(1S,2S,4R,7S)-2-hydroxy-7-(2-methylpropyl)-5,8-dioxo-4-(propan-2-yl)-3-oxa-6,9-diazatricyclo[7.3.0.0ˆ{2,6}]dodecan-4-yl]-6-methyl-6,11diazatetracyclo[7.6.1.0ˆ{2,7}.0ˆ{12,16}]hexadeca-1(16),2,9,12,14-pentaene-4-carboxamide	25614-03-3	Dopamine promoter	3.2	6.71	55	-	+	125	Yes		(28, 51)
Chloroquine	7-chloro-N-[5-(diethylamino)pentan-2-yl]quinolin-4-amine	54-05-7	Immunosuppress-sive and anti-parasitic	4.63	10.32	128	+	+	655	Yes	H, R, D, M	(28, 34, 36, 37, 38, 40, 52, 53, 54, 55)
Chlorpheniramine	[3-(4-chlorophenyl)-3-(pyridin-2-yl) propyl] dimethylamine	132-22-9	Antihistamine	3.38	9.47	101	+	+	147	Yes		(28, 35, 56)
Chlorprothixene	3-[(9Z)-2-chloro-9H-thioxanthen-9-ylidene]propyl}dimethylamine	11-59-7	Antipsychotic	5.18	9.76	122	+	+	7.78	Yes		(28)
Chlorpromazine	[3-(2-chloro-10H-phenothiazin-10-yl)propyl] dimethylamine	50-53-3	Antipsychotic	5.41	9.3	116	+	+	9.01	Yes	R, D	(16, 27, 28, 34, 36, 37, 39, 40)
Cinnarizine	1-(diphenyl methyl)-4-(3-phenylprop-2-en-1-yl) piperazine	298-57-7	Antihistamine	5.77	8.44	105	+	+	40.6	Yes		(28, 57)
Citalopram	1-[3-(dimethylamino)propyl]-1-(4-fluorophenyl)-1,3-dihydro-2-benzofuran-5-carbonitrile	59729-33-8	Antidepressant	3.76	9.78	110	+	+	19.5	Yes	Yes	(12, 27, 28, 34, 35, 36, 37, 40)
Clemastine	(2R)-2-{2-[(1R)-1-(4-chlorophenyl)-1-phenylethoxy] ethyl-1-methylpyrrolidine	14976-57-9	Antihistamine	5.29	9.55	119	+	+	13.21	Yes		(28)
Clenbuterol	4-amino-3,5-dichloro-α-[[(1,1-dimethylethyl)amino]methyl]-benzenemethanol, monohydrochloride	21898-19-1	Muscle relaxer decongestant, bronchodilator	2.94	9.63	101	+	+	7298	Yes		(28)
Clindamycin	(2S,4R)-N-{2-chloro-1-[(2R,3R,4S,5R,6R)-3,4,5-trihydroxy-6-(methylsulfanyl)oxan-2-yl]propyl}-1-methyl-4-propylpyrrolidine-2-carboxamide	18323-44-9	Antibiotic	2.16	7.55	61.7	-	+	ND	Yes	Yes	(12, 28, 36, 40)
Clofazimine	N,5-bis(4-chlorophenyl)-3,5-dihydro-3-(isopropylimino)phenazin-2-amine	2030-63-9	Anti-mycobacterial, anti-inflammatory properties	7.66	9.29	145	+	+	10.8	Yes		(28, 58, 59)
Clomipramine	(3-{14-chloro-2-azatricyclo[9.4.0.0^3^,^8^]pentadeca-1(11), 3,5,7,12,14-hexaen-2-yl}propyl)dimethylamine	303-49-1	Antidepressant	5.04	9.2	104	+	+	17	Yes	Yes	(16, 28, 34, 35, 36, 37, 39)
Cloperastin	1-{2-[(4-chlorophenyl) (phenyl)methoxy] ethyl} piperidine	3703-76-2	Antihistamine	5.11	8.82	104	+	+	40.9	Yes		(28, 30)
Clozapine	8-Chloro-11-(4-methyl-1-piperazinyl)-5H-dibenzo[b,e][1,4]-diazepine	34233-69-7	Antipsychotic	3.23	7.35	64.5	-	+	10.8	Yes	Yes	(16, 28, 34, 39, 40)
Corticosterone	(1S,2R,10S,11S,14S,15S,17S)-17-hydroxy-14-(2-hydroxyacetyl)-2,15-dimethyltetracyclo[8.7.0.0^2^,^7^.0^11^,^15^]heptadec-6-en-5-one	50-22-6	Glucocorticoid	2.09	-0.26	4.4	-	-	163	Yes		(28, 38)
Cyclazosin	1-(4-amino-6,7-dimethoxy-2-quinazolinyl)-4-(2-furanylcarbonyl) decahydroquinoxaline	146929-33-1	Adrenoceptor antagonist	3.4	9.89	109	+	+	30.75	Yes		(28, 37)
Cyclobenzaprine	dimethyl(3-{tricyclo[9.4.0.0^3^,^8^]pentadeca-1(15),3,5, 7,9,11,13-heptaen-2-ylidene}propyl)amine	303-53-7	Muscle relaxant	4.73	8.47	94	+	+	6.24	Yes	Yes	(37)
Cyclopentolate	2-(dimethylamino)ethyl 2-(1-hydroxycyclopentyl)-2-phenylacetate	515-15-2	Anticholinergic	2.32	8.42	76	-	+	32.2	Yes		(28, 60, 61)
Desipramine	(3-{2-azatricyclo [9.4.0.0] pentadeca-1(15),3,5,7,11,13-hexaen-2-yl}propyl)(methyl)amine	58-28-6	Antidepressant	4.02	10.02	117	+	+	25.69	Yes	R	(35, 36, 37, 40, 62)
Dibenzosuberane	10,11-dihydro-5*H*-dibenzo [*a, d*] cycloheptene-833-48-7	833-48-7	Protein inhibitor	4.7	10	122	+	+	346	Yes		(28)
Diphenhydramine	2-[(4-methyl-α-phenyl benzyl) oxy]ethyl (dimethyl) ammonium chloride	147-24-0	Antihistamine	3.27	8.87	89.4	-	+	270	Yes		(28)
Doxepin	dimethyl(3-{9-oxatricyclo[9.4.0.0ˆ[1]]pentadeca-1(15),3,5,7,11,13-hexaen-2-ylidene}propyl)amine	1668-19-5	Psychotropic	4.29	9.76	114	+	+	301	Yes	Yes	(28, 37)
Drofenine	hexahydroadiphenine	548-66-3	Anticholinergic	5.3	9.21	113	+	+	7.29	Yes		(35, 63)
Dutasteride	(1S,2R,7R,10S,11S,14S,15S)-N-[2,5-bis (trifluoromethyl) phenyl]-2,15-dimethyl-5-oxo-6-azatetracyclo[8.7.0.0^2^,^7^.0^11^,^15^]heptadec-3-ene-14-carboxamide	164656-23-9	5α-reductase inhibitor	6.8	2.17	50.9	+	+	1048	Yes		(28)
Encainide	4-methoxy-N-{2-[2-(1-methylpiperidin-2-yl)ethyl] phenyl} benzamide	66778-36-7	Sodium channel blocker	4	9.41	105	+	+	76.09	Yes		(28, 64)
Erythromycin	(3R,4S,5S,6R,7R,9R,11R,12R,13S,14R)-6-{[(2S,3R,4S,6R)-4-(dimethylamino)-3-hydroxy-6-methyloxan-2-yl]oxy}-14-ethyl-7,12,13-trihydroxy-4-{[(2R,4R,5S,6S)-5-hydroxy-4-methoxy-4,6-dimethyloxan-2-yl]oxy}-3,5,7,9,11,13-hexamethyl-1-oxacyclotetradecane-2,10-dione	114-07-8	Bacteriostatic antibiotic	2.37	8.38	75.9		+	117	Yes	R, D	(28, 34, 36, 39, 65)
Etomidate	ethyl 1-[(1R)-1-phenylethyl]-1H-imidazole-5-carboxylate	33125-97-2	Anesthetic	3	4.54	29.6	-	-	1155	Yes		(28, 40)
Fenofibrate	2-[4-(4-chlorobenzoyl)phenoxy]-2-methylpropanoic acid isopropyl ester	49562-28-9	Cholesterol lowering	5.3	-4.9	28.1	-	31.58	2.25	Yes		(28, 66)
Fexofenadine	2-(4-{1-hydroxy-4-[4-(hydroxydiphenylmethyl) piperidin-1-yl]butyl} phenyl)-2-methylpropanoic acid	83799-24-0	Antihistamine	5.02	9.01	106	+	+	179	Yes		(28, 67)
Fipexide	1-(2-[4-chlorophenoxy]acetyl)-4-(3,4-methylenedioxybenzyl)piperazine	34161-24-5	Attention deficit	2.95	6.09	45.7	-	-	17.19	Yes		(28)
Flunarizine	1-[bis(4-fluorophenyl)methyl]-4-[(2E)-3-phenylprop-2-en-1-yl]piperazine	52468-60-7	Calcium entry blocker	5.3	7.6	85.9	-	+	7.49	Yes		(28, 68)
Fluoxetine	methyl({3-phenyl-3-[4-(trifluoromethyl) phenoxy] propyl}) amine	54910-89-3	Antidepressant	4.5	9.8	112	+	+	13.5	Yes	H,R,M	(16, 27, 28, 34, 35, 36, 37, 39, 40, 69, 70)
Flufenamic acid	2-{[3-(trifluoromethyl)phenyl]amino}benzoic acid	530-78-9	Analgesic, anti-inflammatory, antipyretic	5.25	-2.1	32	-	-	177.9	Yes		(28, 71)
Gentisic acid	2,5-dihydroxybenzoic acid sodium salt	4955-90-2	Anti-inflammatory, antioxidant	-1.1	-5.9	34.8	-	-	99.4	Yes		(28, 72)
Hydroxyzine	2-(2-{4-[(4-chlorophenyl) (phenyl) methyl] piperazin-1-yl} ethoxy)ethan-1-ol	68-88-2	Antihistaminic	3.43	7.82	72.9	-	+	63		R	(34, 36, 55, 73)
Imipramine	(3-{2-azatricyclo[9.4.0.0^3^,^8^]pentadeca-1(15),3, 5,7,11, 13-hexaen-2-yl}propyl)dimethylamine	50-49-7	Antidepressant	4.8	9.2	108	+	+	27.6	Yes	R	(16, 28, 34, 35, 36, 37, 40, 74, 75)
Indoramin	N-{1-[2-(1H-indol-3-yl)ethyl]piperidin-4-yl} benzamide	26844-12-2	Antiadrenergic	4.02	9.59	108	+	+	123.9	Yes	R	(28, 36, 40)
Ketoconazole	1-[4-(4-{[2-(2,4-dichlorophenyl)-2-(1H-imidazole-1-ylmethyl)-1,3-dioxolan-4-yl]methoxy}phenyl)piperazin-1-yl]ethan-1-one	65277-42-1	Antifungal	4.35	6.75	64.5	-	+	39	Yes	M	(16, 27, 28, 34, 36, 37, 40)
Ketotifen	4-(1-methylpiperidin-4-ylidene)-4*H*-benzo[4,5]cyclohepta[1,2-*b*]thiophen-9,10-dione	34580-14-8	Antihistamine	2.2	7.15	56	-	+	19.68	Yes		(16, 28, 36, 76)
Lercanidipine	3-{1-[(3,3-diphenylpropyl)(methyl)amino]-2-methylpropan-2-yl} 5-methyl 2,6-dimethyl-4-(3-nitrophenyl)-1,4-dihydropyridine-3,5-dicarboxylate	100427-26-7	Calcium channel blocker	6.4	9.36	129	+	+	37.25	Yes		(28, 77)
Lofepramine	2-[(3-{2-azatricyclo[9.4.0.0ˆ{3,8}]pentadeca-1(15),3,5,7,11,13-hexaen-2-yl}propyl) (methyl) amino]-1-(4-chlorophenyl) ethan-1-one	23047-25-8	Antidepressant	6.11	6.53	80	-	+	13.25	Yes		(28)
Loperamide	4-[4-(4-chlorophenyl)-4-hydroxypiperidin-1-yl]-N,N-dimethyl-2,2-diphenylbutanamide	53179-11-6	Antidiarrheal	4.44	9.41	108	+	+	149.12	Yes		(28)
Loratadine	ethyl 4-{13-chloro-4-azatricyclo[9.4.0.0^3^,^8^]pentadeca-1(11),3(8),4,6,12,14-hexaen-2-ylidene}piperidine-1-carboxylate	79794-75-5	Antihistamine	4.8	4.33	41.8	-	-	8.94	Yes		(16, 28, 36, 37, 40, 78)
Mannitol	(2R,3R,4R,5R)-hexane-1,2,3,4,5,6-hexol	69-65-8	Osmotic diuretic	-2.7	12.3	159	+	+	160	Yes		(28, 38, 79)
Maprotiline	methyl(3-{tetracyclo [6.6.2.0^2^,^7^.0^9^,^14^]hexadeca-2,4,6,9,11, 13-hexaen-1-yl}propyl)amine	10262-69-8	Antidepressant	4.82	10.54	134	+	+	12		R	(34, 35, 36, 79, 80)
Mebeverine	4-{ethyl[1-(4-methoxyphenyl)propan-2-yl]amino}butyl 3,4-dimethoxybenzoate	2743-45-9	Anti-diarrheal	4.6	10.31	127	+	+	417	Yes		(28)
Memantine	3,5-Dimethyl-1-adamantanamine hydrochloride	41100-52-1	NMDA receptor antagonist	3.32	10.7	125	+	+	35.7	Yes		(35, 37, 81, 82)
Methapyrilene	N-[2-(dimethyl amino)ethyl]-N-[(thiophen-2-yl) methyl ]pyridin-2-amine	91-80-5	Antihistamine	2.87	8.85	86.6	-	+	ND	Yes		(28)
Mianserin	5-methyl-2,5 diazatetracyclo [13.4.0.0^2^,^7^.0^8^,^1^³] nonadeca-1(19),8,10,12,15,17-hexaene	24219-97-4	Antidepressant	3.52	6.9	60.3	-	+	29.98	Yes		(28, 33, 36)
Mifepristone	(1S,3aS,3bS,10R,11aS)-10-[4-(dimethylamino )phenyl]-1-hydroxy-11a-methyl-1-(prop-1-yn-1-yl)-1H,2H,3H,3aH, 3bH,4H,5H,7H,8H, 9H,10H,11H,11aH-cyclopenta[a]phenanthren-7-one	84371-65-3	Progesterone blocker	5.3	4.89	52	+	-	27.69	Yes		(28)
Mirtazapine	5-methyl-2,5,19-triazatetracyclo [13.4.0.0^2^,^7^.0^8^,^1^³] nonadeca-1(15),8,10,12,16,18-hexaene	85650-52-8	Antidepressant	2.9	6.67	52.9	-	+	8.7	Yes		(28, 83, 84)
Mitotane	1-chloro-4-[2,2-dichloro-1-(2-chlorophenyl) ethyl] benzene	53-19-0	Chemotherapy	6		36	-	-	132.1	Yes		(28, 85)
Naphazoline	2-(naphthalen-1-ylmethyl)-4,5-dihydro-1H-imidazole	835-31-4	Sympathomimetic	3.44	10.19	115	+	+	6.98	Yes		(35)
Oxolamine citrate	diethyl[2-(3-phenyl-1,2,4-oxadiazol-5-yl)ethyl]amine	959-14-8	Anti-psychotic	2.7	8.96	87.6	-	+	369	Yes		(28, 86)
Oxybutynin	α-phenylcyclohexaneglycolic acid 4-(diethyl amino)-2-butynyl ster hydrochloride	1508-65-2	Anticholinergic	4.36	8.77	95.9	+	+	26.39	Yes		(28)
Pantoprazole	6-(difluoromethoxy)-2-[(3,4-dimethoxypyridin-2-yl) methanesulfinyl] -1H-1,3-benzodiazole	102625-70-7	Proton pump inhibitor	2.11	3.55	17.1	-	-	6.23	Yes		(28, 35, 87)
Paroxetine	(3S,4R)-3-[(2H-1,3-benzodioxol-5-yloxy)methyl]-4-(4-fluorophenyl)piperidine	61869-08-7	Antidepressant	3.1	9.77	105	+	+	5.12		R	(28, 36, 37, 69)
Penfluridol	1-[4,4-bis(4-fluorophenyl)butyl]-4-[4-chloro-3-(trifluoromethyl) phenyl]piperidin-4-ol	26864-56-2	Antipsychotic	6.09	8.96	117	-	+	9.09		Yes	(28, 36, 63)
Perhexiline	2-(2,2-dicyclohexylethyl) piperidine	6621-47-2	Coronary vasodilator	6.2	10.58	150	+	+	11.72	Yes	H,R,M	(16, 28, 34, 35, 36, 40, 88, 89, 90)
Perphenazine	2-{4-[3-(2-chloro-10H-phenothiazin-10-yl)propyl] piperazin-1-yl ethan-1-ol	58-39-9	Antipsychotic	4.2	8.21	85	-	+	3.92	Yes		(28)
Phenacetin	N-(4-ethoxyphenyl) acetamide	62-44-2	Non-steroidal	1.58	-4.2	2.5	-	-	2140	Yes	R	(6, 28, 40, 79, 91)
Pimozide	1-{1-[4,4-bis(4-fluorophenyl)butyl]piperidin-4-yl}-2,3-dihydro-1H-1,3-benzodiazol-2-one	2062-78-4	Antipsychotic	6.36	8.38	111	+	+	10.6	Yes		(28, 92)
Pirenperone	3-[2-[4-(4-Fluorobenzoyl)-1-piperidinyl]ethyl]-2-methyl-4H-pyrido-[1,2-a] pyrimidin-4-one	75444-65-4	Antipsychotic	2.6	8.02	71.1	-	+	232.6	Yes		(28, 93)
Pranlukast	N-[4-oxo-2-(2H-1,2,3,4-tetrazol-5-yl)-4H-chromen-8-yl]-4-(4-phenylbutoxy)benzamide	103177-37-3	Antiasthmatic	4.82	-1.7	23.2	-	-	ND	Yes		(28, 30)
Pridinol	1,1-diphenyl-3-(piperidin-1-yl)propan-1-ol	511-45-5	Muscle relaxant	3.69	9.34	101	+	+	285.4	Yes		(28, 94)
Profenamine	diethyl[1-(10H-phenothiazin-10-yl)propan-2-yl]amine	522-00-9	Antidyskinetic	5.75	9.6	125	+	+	8.7	Yes		(28, 95)
Progesterone	(1S,3aS,3bS,9aR,9bS,11aS)-1-acetyl-9a,11a-dimethyl-1H,2H,3H, 3aH,3bH,4H,5H, 7H,8H,9H,9 aH,9bH, 10H,11H,11aH-cyclopenta [a] phenanthren-7-one	57-83-0	Hormone	3.58	-4.8	12.8	-	-	10.52	Yes		(28, 96)
Promazine	dimethyl[3-(10H-phenothiazin-10-yl)propyl]amine	58-40-2	Antipsychotic	4.55	9.2	105	+	+	39	Yes	R	(28, 34, 35, 40)
Promethazine	dimethyl[1-(10H-phenothiazin-10-yl)propan-2-yl] amine	60-87-7	Antihistamine	4.81	9.05	105	+	+	40.3	Yes	Yes	(28, 37, 40)
Propafenone	1-{2-[2-hydroxy-3-(propylamino)propoxy]phenyl}-3-phenylpropan-1-one	54063-53-5	Antiarrhythmic	3.1	9.63	102	+	+	48.22	Yes		(28, 97)
Proparacaine	2-(diethylamino)ethyl 3-amino-4-propoxybenzoate	499-67-2	Anesthetic	2.5	8.56	79.5	-	+	2469	Yes		(28, 60)
Propranolol	1-(naphthalen-1-yloxy)-3-[(propan-2-yl)amino]propan-2-ol	525-66-6	Antihypertensive	3.48	9.67	106	+	+	49.9	Yes		(1, 28, 34, 35, 36, 37, 40)
Pyrilamine	N-[2-(dimethyl amino)ethyl]-N-[(4-methoxyphenyl) methyl]pyridin-2-amine	91-84-9	Antihistamine	3.27	8.76	87.4	-	+	214	Yes		(28, 98)
Quinacrine	6- chloro-9-(4-diethylamino-1-methylbutylamino)-2-methoxyacridine dihydrochloride	69-05-6	Antimalarial and antibiotic	5.5	10.33	137	+	+	30.13	Yes	R	(28, 34, 35, 36, 37, 40, 99)
Quinine	(R)-[(1S,2S,4S,5R)-5-ethenyl-1-azabicyclo [2.2.2]octan-2-yl](6-methoxyquinolin-4-yl)methanol	130-95-0	Antimalarial	3.34	9.05	93.7	+	+	164		Yes	(36, 40)
Repaglinide	2-ethoxy-4-[2-({3-methyl-1-[2-(1-piperidinyl)phenyl]butyl}amino)-2-oxoethyl]benzoic acid	135062-02-1	Antihyperglycemic	5.9	4.28	53.1	-	+	86.49	Yes		(28, 29)
Retinol	(2E,4E,6E,8E)-3,7-dimethyl-9-(2,6,6-trimethylcyclohex-1-en-1-yl)nona-2,4,6,8-tetraen-1-ol	68-26-8	Vitamin A	5.68	-2.2	37.1	-	-	52.52	Yes		(28, 100)
Ropinirole	4-[2-(dipropylamino)ethyl]-2,3-dihydro-1H-indol-2-one	91374-21-9	Dopamine agonist	3.06	10.17	113	+	+	ND	Yes		(28, 101)
Sertraline	(1S,4S)-4-(3,4-dichlorophenyl)-N-methyl-1,2,3,4-tetrahydronaphthalen-1-amine	79617-96-2	Antidepressant	5.15	9.85	123	+	-	8.6	Yes	Yes	(16, 27, 28, 34, 35, 36, 37, 40)
Spiperone	8-[3-(p-fluor benzoyl)propyl]-1-phenyl-1,3,8-triazaspiro [4.5]decan-4-one	749-02-0	Antipsychotic	3.03	8.89	88.2	-	+	519.2	Yes		(28, 37)
Sulindac	2-[(1Z)-5-fluoro-1-[(4-methanesulfinylphenyl)methylidene]-2-methyl-1H-inden-3-yl]acetic acid	38194-50-2	Nonsteroidal	3.42	-6.7	11.7	-	-	10.93	Yes		(28, 36, 102)
Sulpiride	N-[(1-ethylpyrrolidin-2-yl)methyl]-2-methoxy-5-sulfamoylbenzamide	15676-16-1	Antidepressant, antipsychotic	1.2	8.4	71.8	-	-	ND		Yes	(103)
Suramin	8-{4-methyl-3-[3-({[3-({2-methyl-5-[(4,6,8-trisulfonaphthalen-1yl)carbamoyl]phenyl}carbamoyl)phenyl]carbamoyl}amino)benzamido]benzamido}naphthalene-1,3,5-trisulfonic acid	129-46-4	Antineoplastic	5.58	-6	31.1	-	-	35.55	Yes	R	(28, 104, 105)
Tacrine	1,2,3,4-tetrahydroacridin-9-amine	321-64-2	Cholinesterase inhibitor	2.71	8.95	87.5	-	+	63.5	Yes		(28)
Tamoxifen	(2-{4-[(1Z)-1,2-diphenylbut-1-en-1-yl]phenoxy} ethyl) dimethylamine	10540-29-1	Antiestrogenic	5.93	8.76	112	+	+	7.7	Yes	R	(16, 27, 28, 34, 35, 36, 39)
Tetracaine	2-(dimethylamino)ethyl 4-(butylamino)benzoate	94-24-6	Anaesthetic	3.54	8.42	83.4	-	+	333	Yes		(28)
Thioridazine	10-[2-(1-methylpiperidin-2-yl)ethyl]-2-(methylsulfanyl) -10H-phenothiazine	50-52-2	Antipsychotic	5.9	8.93	115	+	+	38	Yes	R	(16, 28, 36, 40, 106)
Tobramycin	O-[3-amino-3-deoxy-α-D-glucopyranosyl-(1→6)]-O-[2,6-diamino-2,3,6-trideoxy-α- D-ribohexopyranosyl-(1→4)]-2-deoxy-D-streptamine	32986-56-4	Antibiotic	-5.8	9.8	130	+	+	33.03	Yes	R,H	(28, 36, 40, 107, 108, 109)
Trimipramine	(3-{2-azatricyclo[9.4.0.0³,^8^]pentadeca-1(15),3,5,7,11, 13-hexaen-2-yl}-2-methylpropyl)dimethylamine	739-71-9	Antidepressant	4.67	9.42	111	+	+	267.9	Yes		(7, 28, 40)
Triparanol	1-(4-(2-diethylaminoethoxy)phenyl)-1-p-tolyl-2-(4-chlorophenyl)ethanol	78-41-1	Cholesterol synthesis inhibitor	6.2	13.44	219	+	+	7.8	Yes	R, M, Ha	(28, 33, 35, 73, 110, 111, 112, 113)
Vinblastine	methyl (1R,9R,10S,11R,12R,19R)-11-(acetyloxy)-12-ethyl-4-[(13S,15R,17S)-17-ethyl-17-hydroxy-13-(methoxycarbonyl)-1,11-diazatetracyclo [13.3.1.0ˆ[1].0ˆ[1]]nonadeca-4(12),5(10),6,8-tetraen-13-yl]-10-hydroxy-5-methoxy-8-methyl-8,16-diazapentacyclo [10.6.1.0ˆ[1].0ˆ[1] .0ˆ[3]] nonadeca-2,4,6,13-tetraene-10-carboxylate	865-21-4	Chemotherapeutic	3.7	8.86	92.2	+	+	2123	Yes		(28, 97)
Warfarin	4-hydroxy-3-(3-oxo-1-phenylbutyl)-2H-chromen-2-one	81-81-2	Anticoagulant	2.41	-6.6	49.4	-	-	94	Yes		(28, 114)
Yohimbine	methyl (1S,15R,18S,19R,20S)-18-hydroxy-3,13-diazapentacyclo [11.8.0.0^2^,^10^.0^4^,^9^.0^15^,^20^]henicosa-2(10),4,6,8-tetraene-19-carboxylate	146-48-5	Alpha adrenergic antagonist	2.73	7.65	66	-	+	200	Yes		(28)
Zafirlukast	cyclopentyl N-[3-({2-methoxy-4-[(2-methylbenzenesulfonyl) carbamoyl]phenyl}methyl)-1-methyl-1H-indol-5-yl]carbamate	107753-78-6	Leukotriene receptor antagonist	5.4	-1.1	29.2	-	-	3.1	Yes		(35)

The generic name, International Union of Pure and Applied Chemistry (IUPAC) designation, chemical abstracts registry (CAS) number, and clinical indication are provided. Chemical properties, including ClogP and pKa (basic) were obtained from the Pubchem and chEMBL databases of bioactive molecules and used to calculate the Ploemen value for each compound. By convention, negative pKa values were assigned a value of 0 for calculation of the Ploemen number and would be predicted negative based on pKa values of less than 8 or 6 for the Ploemen and modified Ploemen models respectively (Table 3). The species for in vivo data are designated as human (H), dog (D), rat (R), mouse (M), and hamster (Ha). LPLA_2_ IC_50_ denotes the concentration at which 50% of the LPLA_2_ dependent 1-O-acyl N-acetylsphingosine synthase activity is observed.

**Table 2:** Test compounds not previously associated with phospholipidosis

Generic Name	UPAC Designation	CAS Number	Indication	ClogP	pKa (basic)	Ploemen Value	Pred Ploemen	Pred Mod Ploemen	LPLA2 IC50 (μM)
Amisulpride	4-amino-N-[(1-ethyl-2-pyrrolidinyl)methyl]-5-(ethylsulfonyl)-2-methoxybenzamide	71675-85-9	antipsychotic	1.5	7.05	52	-	-	1915
Allopurinol	3-(4*H*-1,2,4-triazol-4-yl)-1*H*-pyrazole-4-carboxamide	315-30-0	xanthine oxidase inhibitor	-0.55	2.57	6.9	-	-	ND
Atovaquone	2-hydroxy-3-[(1r,4r)-4-(4-chlorophenyl) cyclohexyl] -1,4-dihydronaphthalene-1,4-dione	95233-18-4	anti-pneumocystis, anti-malarial	1.59	8.16	69.1	-	-	ND
Atropine	(1R,3R,5S)-8-methyl-8-azabicyclo[3.2.1]octan-3-yl 3-hydroxy-2-phenylpropanoate	51-55-8	antimuscarinic	1.83	9.39	91.5	-	-	1874
Azaperone	1-(4-fluorophenyl)-4-[4-(pyridin-2-yl)piperazin-1-yl] butan-1-one	1649-18-9	tranquilizer	2.73	7.16	58.7	-	+	ND
Benztropine	(1R,3R,5S)-3-(diphenylmethoxy)-8-methyl-8-azabicyclo[3.2.1]octane	86-13-5	antitremor	4.27	9.54	109	+	+	58.97
18 Beta-glycyrrhetinic acid	3β-hydroxy-11-oxo-18β,20β-olean-12-en-29-oic acid	471-53-4	anti-inflammatory, antioxidant	3.7	16	270	+	+	2432
Butenafine	[(4-tert-butylphenyl)methyl](methyl)(naphthalen-1-ylmethyl)amine	101828-21-1	antifungal	5.85	9.23	119	+	+	41.2
Carbamazepine	2-azatricyclo[9.4.0.0^3^,^8^]pentadeca-1(15),3,5,7,9,11,13-heptaene-2-carboxamide	298-46-4	anticonvulsant	2.77	-3.8	7.7	-	-	105.6
Clomifene	2-[4-(2-chloro-1,2-diphenylethenyl) phenoxy]ethyl} diethylamine	911-45-5	estrogen agonist	7.2	9.31	139	+	+	12.86
Clonidine	N-(2,6-dichlorophenyl)-4,5-dihydro-1H-imidazole-2-amine	4205-90-7	antihypertensive	1.59	8.16	69.1	-	-	ND
Cloricromen	8-chloro-3-(2-diethylaminoethyl)-7-ethoxycarbonylmethoxy-4-methylcoumarin hydrochloride	74697-28-2	platelet aggregation inhibitor	3.97	9.1	98.6	-	+	285
Conessin	(3β)-N,N-dimethyl-con-5-enin-3-amine	546-06-5	antihistamine	4.9	5	49	-	-	31.28
Desloratadine	13-chloro-2-(piperidin-4-ylidene)-4-azatricyclo [9.4.0.0^3^,^8^] pentadeca-1(11),3(8),4,6,12,14-hexaene	100643-71-8	antihistamine	3.48	9.73	107	+	+	8.36
D-(+)-glucose	(2R,3S,4R,5R)-2,3,4,5,6-pentahydroxyhexanal	50-99-7	monosaccharide	-2.4	-3	14.8	-	-	ND
Diclofenac	(2-[(2,6-dichlorophenyl)amino] benzene acetic acid sodium salt-15307-79-6)	15307-79-6	nonsteroidal anti inflammatory	4.51	-2.1	20.3	-	-	7.2
5,7 Dichloro-8-hydroxy-2-methyl quinolone	5,7-Dichloro-2-methyl-8-quinolinol	72-80-0	antioxidant	3.61	4	29	-	-	1179
Dilazep	3-{4-[3-(3,4,5-trimethoxybenzoyloxy)propyl]-1,4-diazepan-1-yl} propyl 3,4,5-trimethoxybenzoate	35898-87-4	adenosine uptake inhibitor	3.21	9.54	101	+	+	59.76
1,7-Dimethylxanthine	1,7-Dimethyl-1*H*-purine-2,6-dione	611-59-6	stimulant	-0.8	13.5	183	-	-	ND
Disopyramide	4-[bis(propan-2-yl)amino]-2-phenyl-2-(pyridin-2-yl) butanamide	3737-09-5	antiarrhythmic	2.58	10.31	115	+	+	ND
Fenspiride	8-(2-phenylethyl)-1-oxa-3,8-diazaspiro[4.5]decan-2-one	5053-08-7	anti-inflammatory	1.81	9.37	91.1	-	-	ND
Fosinopril	(2S,4S)-4-cyclohexyl-1-{2-[(R)-[(1S)-2-methyl-1-(propanoyloxy)propoxy](4-phenylbutyl) phosphoryl] acetyl} pyrrolidine-2-carboxylic acid	98048-97-6	Angiotensin-converting enzyme inhibitor	5.62	-4.4	31.6	-		0.181
Fosinoprilat	(2S,4S)-4-cyclohexyl-1-{2-[hydroxy(4-phenylbutyl)phosphoryl]acetyl}pyrrolidine-2-carboxylic acid	95399-71-6	active fosinopril metabolite	3.7	-4.7	35.8	-	-	5.764
Fulvestrant	(1S,3aS,3bR,4R,9bS,11aS)-11a-methyl-4-[9-(4,4,5,5,5-pentafluoropentanesulfinyl)nonyl]-1H,2H,3H,3aH,3bH,4H,5H,9bH,10H,11H,11aH-cyclopenta[a]phenanthrene-1,7-diol	129453-61-8	chemotherapeutic	6.54	-0.88	42.8	-	-	61.85
Fusidic acid	(3α,4α,5α,8α,9β,11α,13α,14, 16β,17Z)-16-acetoxy-3,11-dihydroxy-4,8,14-trimethyl-18-norcholesta-17,24-dien-21-oic acid	6990-06-3	antibiotic	4.97	-0.2	24.7	-	-	231
Gabapentin	2-[1-(amino methyl) cyclohexyl]acetic acid	60142-96-3	antiepileptic	-1.9	9.91	102	-	-	37.37
Harmine	7-methoxy-1-methyl-9H-pyrido[3,4-b]indole	442-51-3	central nervous system stimulant.	2.61	6.46	48.5	-	-	633
Hydralazine	1-hydrazinylphthalazine	86-54-4	antihypertensive	1	6.4	41	-	-	100
Hydrocortisone	(11β)-11,17,21-trihydroxypregn-4-ene-3,20-dione	50-23-7	glucocorticoid	1.61	-2.8	10.4	-	-	402
6-Hydroxy-dopamine	2,4,5-rihydroxyphenethylamine hydrochloride	28094-15-7	neurotoxin	0.26	9.85	97.1	-	-	88.02
3-Hydroxy-tyramine hydrochloride	2-(3,4-dihydroxyphenyl)ethylamine hydrochloride, 3,4-dihydroxyphenethylamine hydrochloride	62-31-7	catecholamine neurotransmitter	-0.91	9.27	86.9	-	-	56.08
Imiquimod	1-(2-methylpropyl)-1H-imidazo[4,5-c]quinolin-4-amine	99011-02-6	topical immunomodulatory	2.83	5.01	33.1	-	+	160
Isoxsuprine	4-[(1S,2R)-1-hydroxy-2-{[(2R)-1-phenoxypropan-2-yl]amino}propyl]phenol	395-28-8	vasodilator	2.06	9	85.2	-	+	ND
Lidocaine	2-diethylamino-*N*-(2,6-dimethylphenyl)acetamide	137-58-6	antiarrhythmic	1.8	7.75	63.3	-	-	ND
L-leucine	(2S)-2-amino-4-methylpentanoic acid	61-90-5	amino acid	-1.52	9.52	92.9	-	-	ND
Mebhydrolin	5-benzyl-2-methyl-1H,2H,3H,4H,5H-pyrido[4,3-b]indole	524-81-2	antihistamine	3.5	6.7	57.1	-	+	146
Meclofenamic acid	2-[(2,6-dichloro-3-methylphenyl)amino]benzoic acid	644-62-2	nonsteroidal	5.11	-3.6	39.1	-	-	ND
Melatonin	*N*-acetyl-5-methoxytryptamine	73-31-4	antigonadotrope	1.42	-1.6	4.6	-	-	38.03
Mibefradil	(1S,2S)-2-(2-{[3-(1H-1,3-benzodiazol-2-yl)propyl] (methyl)amino}ethyl)-6-fluoro-1-(propan-2-yl)-1,2,3,4-tetrahydronaphthalen-2-yl 2-methoxyacetate	116644-53-2	calcium channel blocker	5.34	9.82	124	+	+	12.92
Naproxen	(2S)-2-(6-methoxynaphthalen-2-yl) propanoic acid	22204-53-1	nonsteroidal	3.18	-4.8	33.2	-	-	1102
Orphenadrine	dimethyl({2-[(2-methylphenyl) (phenyl) methoxy] ethyl}) amine	83-98-7	muscle relaxant	3.77	8.87	92.9	+	+	259.3
Phenytoin	5,5-diphenyl-2,4-imidazolidinedione, 5,5-Diphenylhydantoin sodium salt	630-93-3	anticonvulsant	2.47	-9	6.1	-	-	60.05
Pipamperone	1′-[4-(4-fluorophenyl)-4-oxobutyl]-[1,4′-bipiperidine]-4′-carboxamide	1893-33-0	antipsychotic	2.32	8.96	85.7	-	+	250
PP 06424439	(3R)-1-[2-[1-(4-chloro-1H-pyrazol-1-yl)cyclopropyl]-3H-imidazo[4,5-b]pyridin-5-yl]-3-piperidinyl]-1-pyrrolidinyl-methanone	1469284-79-4	triglyceride and cholesterol lowering	2.7	4.1	24.1	-	-	8217
Prochlorperazine	2-chloro-10-[3-(4-methylpiperazin-1-yl)propyl]-10H-phenothiazine	58-38-8	antipsychotic	4.88	8.39	94.2	+	+	42.41
Procyclidine	1-cyclohexyl-1-phenyl-3-(pyrrolidin-1-yl)propan-1-ol	77-37-2	anticholinergic	4.7	13.84	214	+	+	1470
Ritanserin	6-(2-{4-[bis(4-fluorophenyl)methylidene]piperidin-1-yl}ethyl)-7-methyl-5H-[1,3]thiazolo[3,2-a]pyrimidin-5-one	87051-43-2	serotonin receptor agonist	5.02	8	89.2	-	+	13.39
Rolipram	4-[3-(cyclopentyloxy)-4-methoxyphenyl]pyrrolidin-2-one	61413-54-5	antidepressant	2.15	-1.9	9.9	-	-	300
SB222200	(*S*)-3-methyl-2-phenyl-N-(1-phenylpropyl)-4-quinolinecarboxamide-174635-69-9	174635-69-9	antihistamine	2.17	9.57	98.2	+	+	14.94
S-methyl-isothiourea	methyl carbamimidothioate	867-44-7	iNOS inhibitor	1.47	9.83	98.8	-	-	ND
Suloctidil	(1R,2S)-2-(octylamino)-1-[4-(propan-2-ylsulfanyl)phenyl]propan-1-ol	54767-75-8	vasodilator	5.54	9.76	126	+	+	6.82
Trifluoperazine	10-[3-(4-methylpiperazin-1-yl)propyl]-2-(trifluoromethyl) -10H-phenothiazine	117-89-5	antipsychotic, antiemetic	4.87	8.39	94.1	+	+	6.87
Uridine	1-[(2R,3R,4S,5R)-3,4-dihydroxy-5-(hydroxymethyl) oxolan-2-yl]-1,2,3,4-tetrahydropyrimidine-2,4-dione	58-96-8	pyrimidine analog	-1.98	-3	3.9	-	-	ND
Xylometazoline	2-[(4-tert-butyl-2,6-dimethylphenyl)methyl]-4,5-dihydro-1H-imidazole	526-36-3	decongestant	3.2	10.29	129	+	+	44.32

The generic name, International Union of Pure and Applied Chemistry (IUPAC) designation, chemical abstracts registry (CAS) number, and clinical indication are provided. Chemical properties, including ClogP and pKa (basic) were obtained from the Pubchem and chEMBL database of bioactive molecules and used to calculate the Ploemen value for each compound. By convention, negative pKa values were assigned a value of 0 for calculation of the Ploemen number and would be predicted negative based on pKa values of less than 8 or 6 for the Ploemen and modified Ploemen models respectively. LPLA_2_ IC_50_ denotes the concentration at which 50% of the LPLA_2_ dependent 1-O-acyl N-acetylsphingosine synthase activity is observed.

Two primary physical properties of a drug have been used to predict whether a compound may be lysosomotropic. These are the ClogP and pKa (basic). ClogP, a measure of partitioning between octanol and water, predictive of transport independent distribution across cell membranes. pKa (basic) is a determinant of the protonation of an amine at lysosomal pH. ClogP and pKa (basic) were employed by Ploemen and colleagues to generate *an* in silico model that is predictive of phospholipidosis (Table 3) (115). In a subsequent paper, a modification was proposed to improve the positive and negative predictive value of the model (11). In contrast, the assay used for the measurement of LPLA_2_ activity is cell-free and thus not dependent on the ability of a particular compound to enter a target cell and distribute into late endosomes or lysosomes. A comparison between the physical properties and inhibitory effects on LPLA_2_ of the compounds tested was therefore assessed as independent variables.

**Table 3:** The Ploemen and modified Ploemen criteria for prediction of phospholipidosis

	Ploemen Model	Modified Ploemen Model
Predicted positive	If (pKa-basic)2 + (ClogP)2 ≥ 90, provided that pKa ≥ 8 and ClogP ≥ 1	If (pKa-basic)2 + (ClogP)2 ≥ 50, provided that pKa ≥ 6 and ClogP ≥ 2
Predicted negative	When result < 90 or pKa < 8 or ClogP < 1	When result < 50 or pKa < 6 or ClogP < 2

**Fig. 1:**
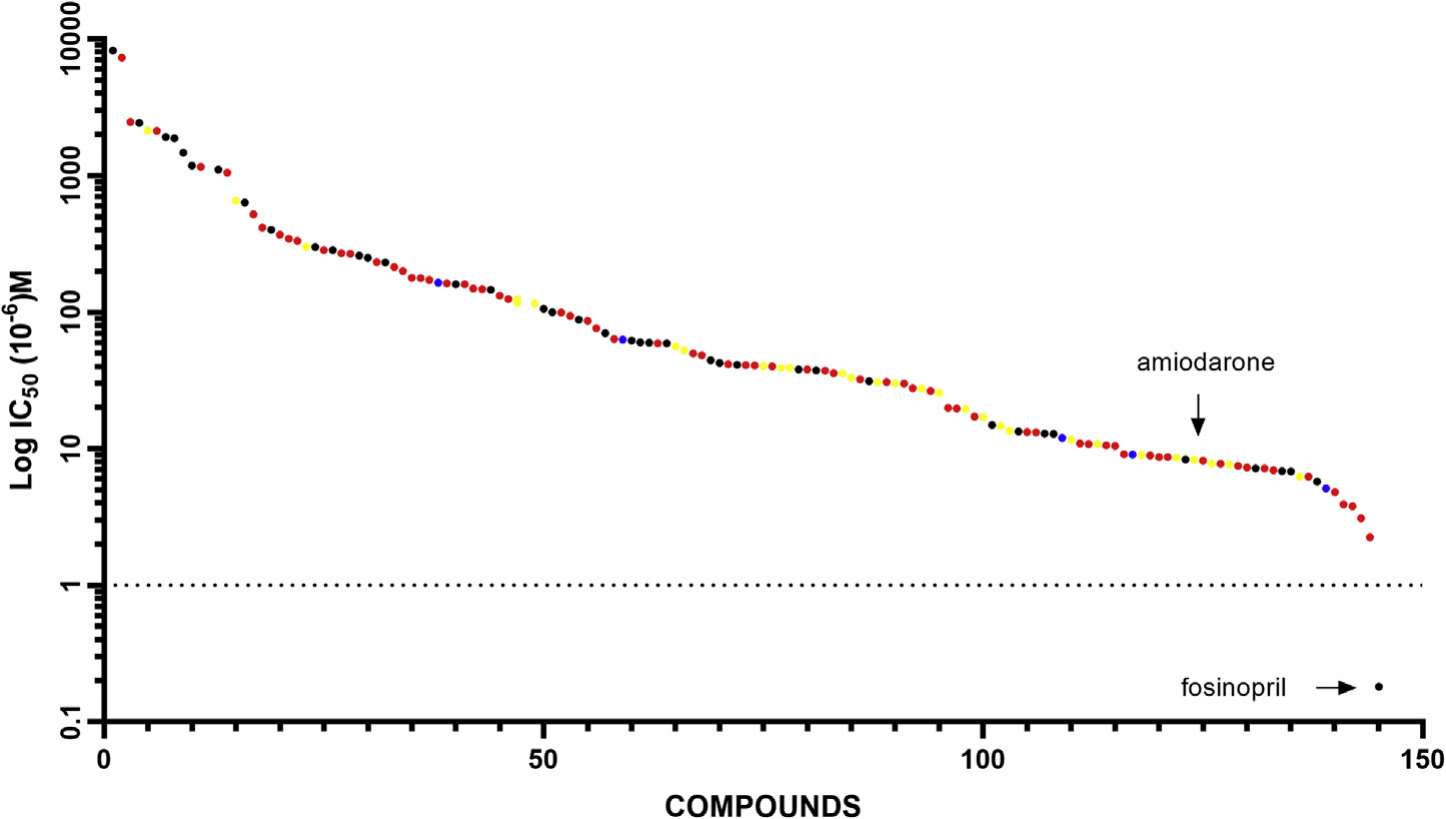
The range of LPLA_2_ inhibition by all compounds studied. The measured IC_50_s for inhibition of LPLA_2_ in the cell-free assay are plotted. Compounds from Table 1 in which phospholipidosis has been reported are denoted by in vitro studies (red circles), in vivo studies (blue circles), or both in vitro and in vivo studies (yellow circles). Compounds studied in which no prior reports of phospholipidosis are denoted by black circles.

Under these assay conditions, 112 compounds from the entire library of known phospholipidotic and control compounds inhibited LPLA_2_ acyl transferase activity at IC_50_ values less than 250 μM. Twenty-eight compounds inhibited with IC_50_s greater than 250 μM, and 19 compounds showed no inhibition (Fig. 1). Surprisingly, 35 compounds not previously reported to cause phospholipidosis inhibited LPLA_2_ with 22 compounds inhibiting at IC_50_s of 250 μM or less (black circles). The measured IC_50_s were continuous between 3.8 μM and 2 mM. The compounds assayed did not segregate based on therapeutic use or whether phospholipidosis had been reported as a result of in vitro (red circles), in vivo (blue circles) or both in vitro and in vivo studies (yellow circles). The most potent inhibitor among this group was fosinopril, an angiotensin-converting enzyme inhibitor not previously reported to cause phospholipidosis.

**Fig. 2:**
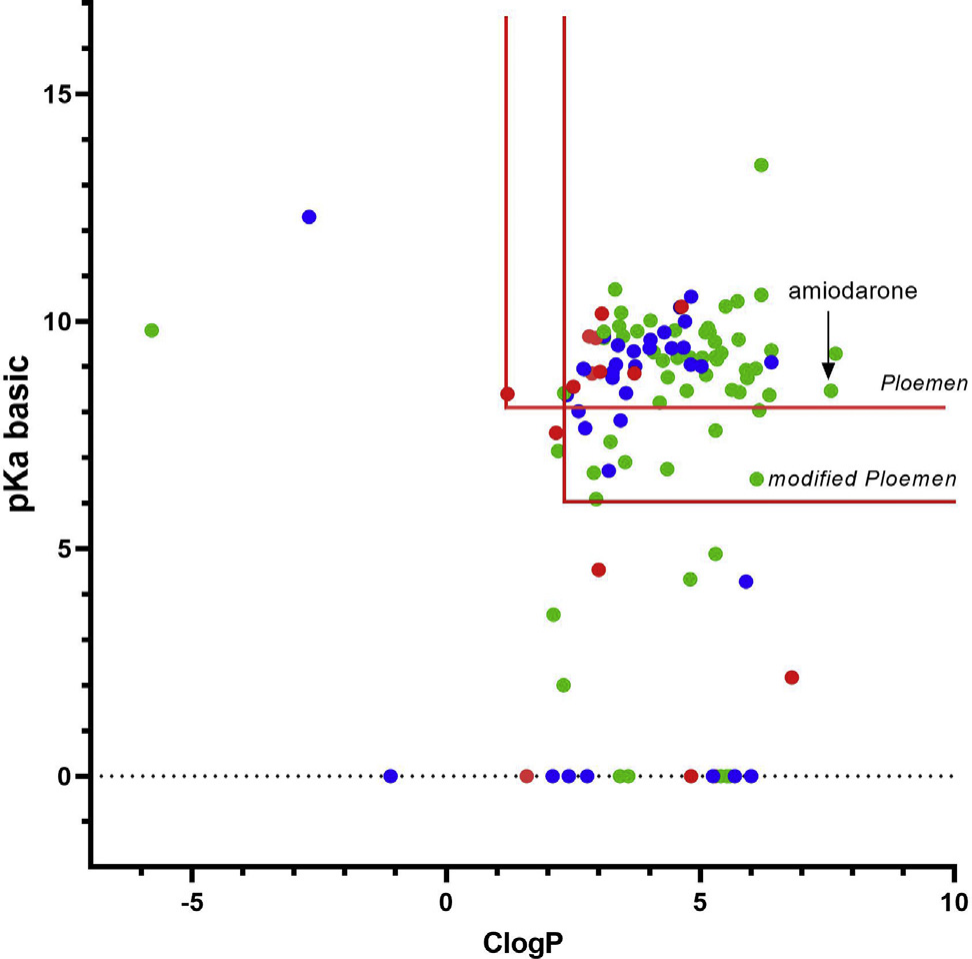
LPLA_2_ inhibition in relation to the physical properties of compounds previously associated with phospholipidosis. The test compounds listed in Table 1 are graphed in relation to pKa (basic) and ClogP. The exclusion limits of the Ploemen and modified Ploemen models are delineated by the red lines. The IC_50_s for LPLA_2_-dependent 1-O-acyl N-acetylsphingosine synthase activity are indicated as follows: greater than 1 mM (red circles), less than 100 μM (green circles), greater than 100 μM and less than 1 μM (blue circles).

The library of compounds previously reported to cause phospholipidosis and assayed for LPLA_2_ inhibition were plotted based on their calculated ClogP and pKa basic values (Fig. 2). Following the convention employed in the Ploemen study, negative pKa basic values were assigned a value of 0 for purposes of calculating a Ploemen value. Eight compounds inhibited LPLA_2_ at concentrations greater than 500 μM. Three compounds (proparacaine, vinblastine, and clenbuterol) inhibited LPLA_2_ at millimolar concentrations, and two compounds, spiperone and chloroquine, inhibited LPLA_2_ with IC_50_s slightly greater than 500 μM. Four compounds (betaxolol, methapyrilene, ropinirole, and sulpiride) had no inhibitory activity against LPLA_2_ and could thus be considered to be true false-negatives for predicting phospholipidosis.

Importantly, 23 compounds in the library reported to cause phospholipidosis did not meet either the Ploemen or modified Ploemen criteria. Of these 23 compounds, 17 inhibited LPLA_2_, 9 of these compounds having IC_50_ values less than 50 μM. Thus in this limited library of compounds previously reported to cause phospholipidosis, LPLA_2_ inhibition was observed for almost three-quarters of the drugs that would have been considered false-negatives by the Ploemen or modified Ploemen criteria.

**Fig. 3:**
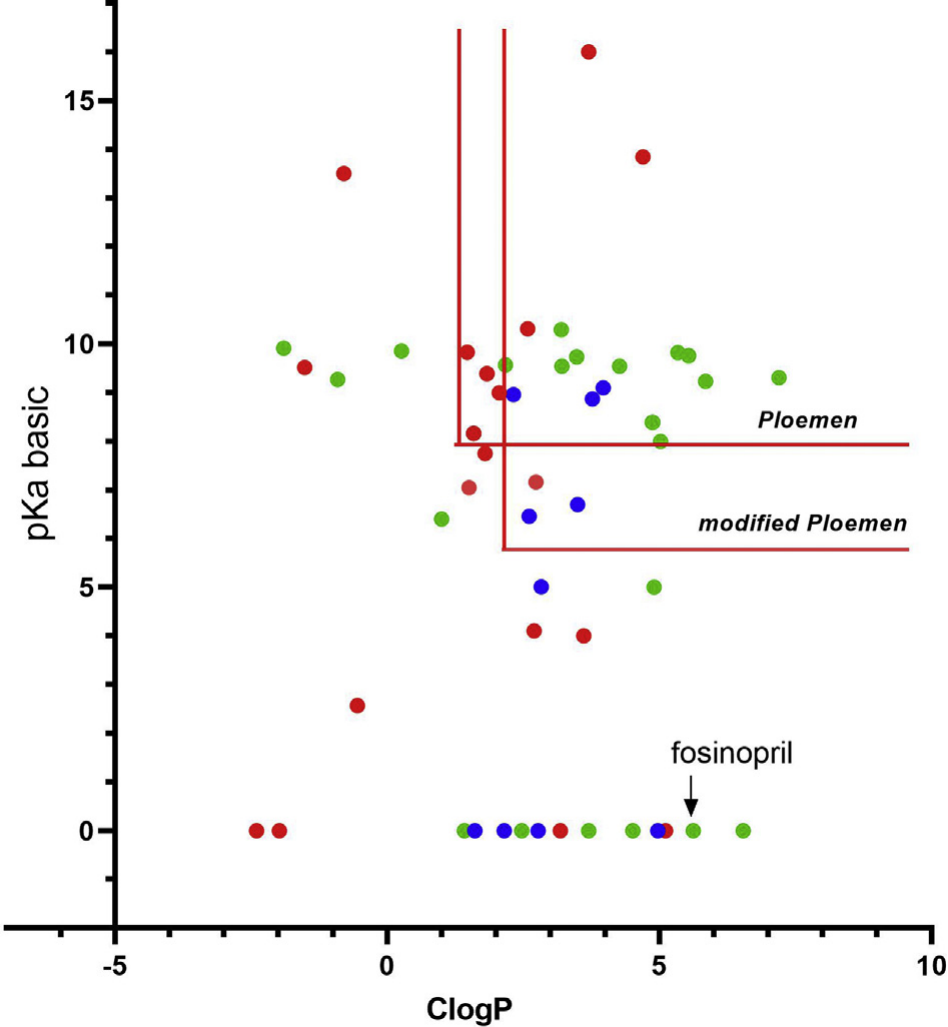
LPLA_2_ inhibition in relation to the physical properties of compounds not previously associated with phospholipidosis. The test compounds listed in Table 2 are graphed in relation to pKa (basic) and ClogP. The exclusion limits of the Ploemen and modified Ploemen models are delineated by the red lines. LPLA_2_ IC_50_s for LPLA_2_-dependent 1-O-acyl N-acetylsphingosine synthase activity are indicated as follows: greater than 1 mM (red circles), less than 100 μM (green circles), greater than 100 μM and less than 1 μM blue circles.

The second library consisting of compounds not reported to cause phospholipidosis was similarly graphed (Fig. 3). Thirty of 55 compounds inhibited LPLA_2_ at IC_50_ values less than 250 μM, and 15 of these compounds inhibited LPLA_2_ at less than 50 μM. Only half or 15 of the 30 inhibitors would have been identified by the modified Ploemen criteria. An in vitro assay using the LipidTOX red detection reagent was used to determine whether exposure of cells to these compounds was associated with lysosomal phospholipid accumulation (Table 4). This assay was validated using fosinopril, the most potent inhibitor of LPLA_2_ activity (supplemental Figs. S1 and S2). In addition, none of the 163 compounds shifted the melting temperature of LPLA_2_ more than 2°C when assayed for thermal stability, consistent with a lack of direct binding of any compound to the phospholipase (supplemental Table 1). In contrast the fluorophosphonate inhibitors, isopropyl dodec-11-enyl fluorophosphonate and methyl arachidonyl fluorophosphonate, covalently bind to the catalytic serine of LPLA_2_ and increase the melting temperature by 10 and 12°C, respectively (22).

**Table 4:** Screening phospholipidosis assay

Drug	Ratio (0.32 μM)	Ratio (15 μM)
Allopurinol	0.13	0.37
Amisulpride	0.08	0.08
Atropine	0.04	0.16
Azaperone	0.16	0.43
Benztropine	0.13	0.68
Benzbromarone	0	0.5
18 Beta-glycyrrhetinic acid	0.16	0.4
Butenafine	0.16	0.31
Carbamazepine	0.12	0.18
Clofazimine	0.11	0.15
Clonidine	0.18	0.25
Clomifene	0.09	0.38
Cloricromen	0.1	0.1
Conessin	0.01	1.08
Corticosterone	0.12	0.28
Desloratadine	0.14	1.13
Diclofenac	0.22	0.49
5,7 Dichloro-8-hydroxy-2-methyl quinolone	0.17	0.29
Dilazep	0.37	1.17
1,7-Dimethylxanthine	0.16	0.55
Disopyramide	0.16	0.57
Encainide	0.09	0.23
Fosinopropil	0.18	0.67
Fosinoprilat	0.07	0.19
Fenspiride	0.07	0.07
Fulvestrant	0.2	0.37
Fuscidic acid	0.13	0.23
Gabapentin	0.07	0.25
Harmine	0	0.11
Hydrocortisone	0.12	0.15
6-Hydroxydopamine	0.2	0.93
3-Hydroxytyramine hydrochloride	0.14	0.91
Hydralazine	0.1	0.6
Imiquimod	0.07	0.07
Isoxsuprine	0.16	1.22
Lidocaine	0.16	0.45
Mebhydrolin	0.12	2.54
Meclofenamic acid	0.18	0.52
S-methylisothiourea	0.15	0.38
Melatonin	0.15	0.26
Mibefradil	0.02	1.21
Naproxen	0.07	0.07
Orphenadrine	0.17	0.29
Paroxetine	0.03	1.11
Penfluridol	0.01	0.55
Phenytoin	0.23	0.38
Pipamperone	0.17	0.37
pp 06424439	0.15	0.21
Prochlorperazine	0.16	0.81
Procyclidine	0.16	1.14
Quinine	0.12	0.34
Ritanserin	0.07	0.19
Rolipram	0.11	0.19
sb222200	0.03	0.34
Suloctidil	0.14	0.66
Trifluoperazine	0.24	0.81
Xylometazoline	0.14	0.16
Amiodarone	0.32	1.64

Each compound was assayed at either 32 or 15 μM. The fluorescence ratio denotes LipidTOX Red Phospholipidosis detection (549–615 nm emission) to NucBlue Live detection (410–480 nm emission).

**Fig. 4:**
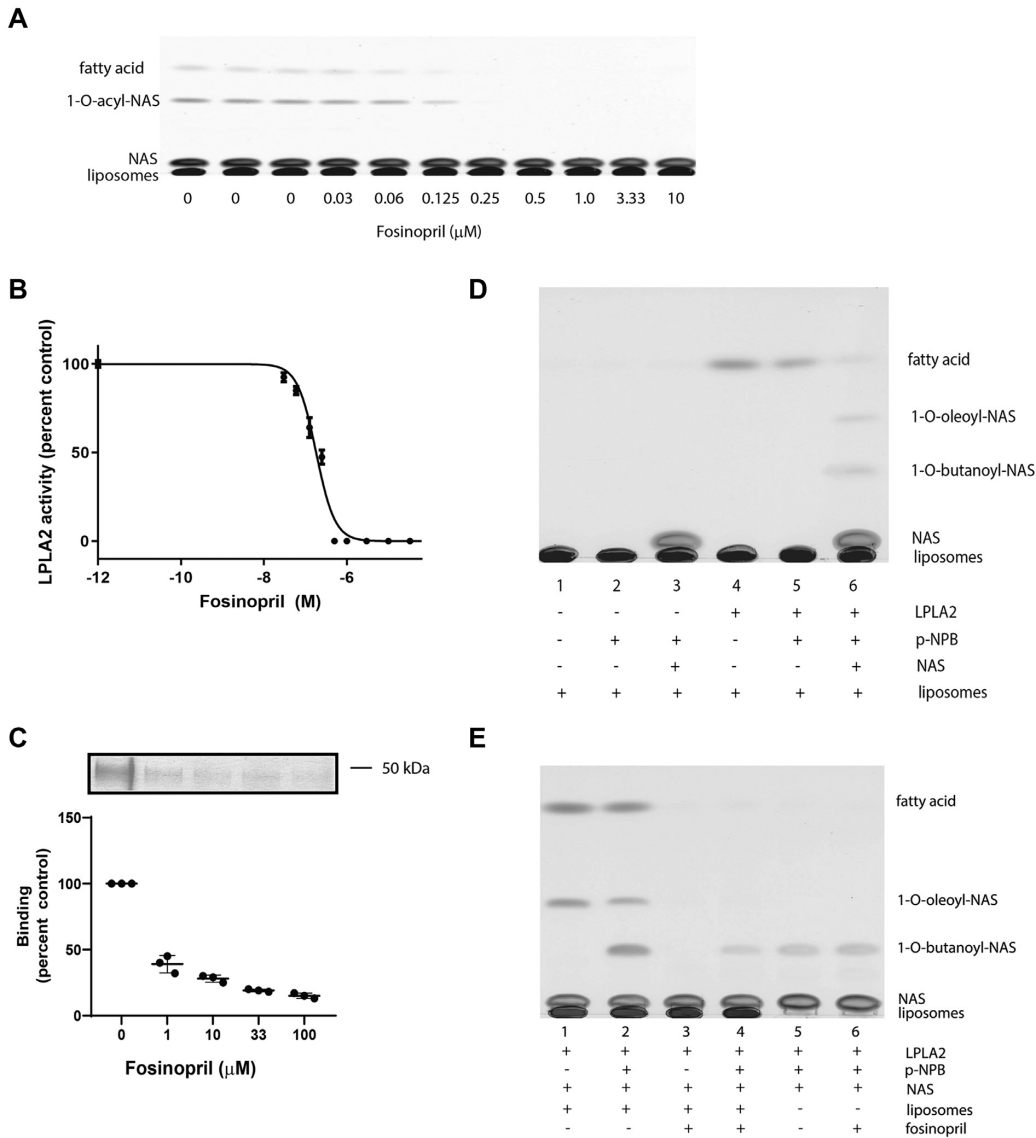
Inhibition of LPLA_2_ by fosinopril. A: Thin layer chromatography from the LPLA_2_ assay in the presence of fosinopril. The reaction products include free fatty acid and 1-O-acyl-N-acetyl-ceramide (1-O-acyl-NAS). B: LPLA_2_ activity in the presence of fosinopril as a percent of the control assay run in the absence of fosinopril. C: Cosedimentation of liposomes and LPLA_2_ in the presence or absence of fosinopril. Liposomes consisting of DOPC/ sulfatide (10:1, molar ratio, 127 μM total) were incubated with 5 μg of LPLA_2_ and different concentrations of fosinopril in 500 μl of 50 mM sodium citrate pH 4.5 for 30 min on ice. The reaction mixture was then centrifuged for 1 h at 150,000 *g* at 4°C. The resulting precipitate was rinsed with cold 50 mM sodium citrate pH 4.5 and dissolved with 40 μl of SDS-PAGE sample buffer. The sample was separated by using 10% SDS-PAGE. Following electrophoresis, LPLA_2_ was detected with Simply Blue. Band quantification was performed with the *Image J* software I1.651j8. D: LPLA_2_ transacylase activity against comparing DOPC to p-NPB as substrates. Liposomes containing DOPC-sulfatide (10:1 M ratio) were incubated with recombinant LPLA_2_ (30 ng/ml) with or without p-NPB (200 μM) in the presence or absence of 10 μM NAS at 37 degrees C in 500 μl Na-citrate buffer (50 mM, pH 4.5). E: LPLA_2_ transacylation activity toward p-NPB comparing liposomes to a monodispersed substrate. Fosinopril (250 nM) was present in lanes 5 and 6. The reactions as detailed in panels E and F were terminated by the addition of 3 ml chloroform/methanol (2/1, v/v), followed by 0.3 ml of 9% (w/v) NaCl. After centrifugation for 7 min at 1,800 *g*, the resulting lower layer was transferred to new tube and dried under stream of nitrogen gas. The dried lipid was dissolved in 40 μl of chloroform/methanol (2/1, v/v) and applied to HPTLC plates. HPTLC plates were run in chloroform/acetic acid (9/1, v/v). The plates were dried and soaked in 8% (w/v) CuSO_4_.5H_2_O, 6.8% (v/v) H_3_PO_4_, and 32% (v/v) methanol and then charred for 15 min in an oven at 150 ˚C. Scanned plates were analyzed by NIH ImageJ 1.651j8 (National Institutes of Health).

Fosinopril is an angiotensin-converting enzyme inhibitor not previously known to cause phospholipidosis. Fosinopril inhibited LPLA_2_ activity at an IC_50_ of 180 nM, considerably lower than that observed for any other compound (Fig. 4A and B). The basis for LPLA_2_ inhibition by this compound was therefore studied in greater detail. We had previously shown that the inhibition of electrostatic binding of liposomes to LPLA_2_ could be measured by loss of cosedimentation. Fosinopril partially inhibited the cosedimentation of liposomes and recombinant LPLA_2_ when centrifugation was performed at 150,000 *g* (Fig. 4C). As was previously reported with amiodarone (25), no inhibition of the soluble esterase activity of LPLA_2_ was observed in the presence of fosinopril. The transacylase activity of LPLA_2_ toward p-NPB as substrate was first confirmed by the formation of 1-O-butanoyl-N-acetylsphnigosine when present in the fully constituted LPLA_2_ assay (Fig. 4D). The formation of 1-O-butanoyl-N-acetylsphingosine was also observed when LPLA_2_ activity was assayed only in the presence of LPLA_2_, N-acetylsphingosine, and p-NPB as a monodispersion (Fig. 4E) consistent with accessibility of the substrate and acceptor within the catalytic domain of LPLA_2_. However, in the presence of 250 nM fosinopril, no inhibition of 1-O-butanoyl-N-acetylsphingosine formation was observed. This is consistent with the absence of direct inhibition of LPLA_2_ by this drug.

Compared with untreated controls, 325 nM fosinopril significantly increased the number of LipidTOX Red particles as assessed by fluorescence microscopy (supplemental Fig. S1). Quantification of particles revealed an approximate 20-fold increase in particle number in fosinopril-treated cells compared with controls (supplemental Fig. S2), which was similar to that observed with amiodarone at the same concentration. Concomitant increases in percent area and mean fluorescence intensity (MFI) were also documented.

Fosinopril is a prodrug for the active metabolite fosinoprilat. The measured IC_50_ value for fosinoprilat (5.8 μM) was more than 50 times greater than that observed for fosinopril (0.18 μM). However, fosinoprilat also generated a positive signal in the LipidTOX assay (Table 4). Thus metabolism of fosinopril to its active metabolite cannot explain the previously reported absence of phospholipidosis in this case.

## Discussion

There are three important findings in this study. First, inhibition of LPLA_2_, as measured by a decrease in 1-O-acylceramide formation in a cell-free assay, is observed in the presence of most of 110 drugs studied previously reported to cause phospholipidosis. None of the drugs assayed shifted the melting temperature of LPLA_2_ consistent with the absence of the direct binding of any compound to LPLA_2_. Thus the inhibition of LPLA_2_ activity by these compounds occurs within a concentration range and likely by a mechanism similar to amiodarone, namely interference with the electrostatic charge interaction between cationic residues in the lipid-binding domain of LPLA_2_ and anionic phospholipid head groups. This mechanism, however, would not explain the inhibition of LPLA_2_ by compounds that cause phospholipidosis but lack a functional group that would be protonated at lysosomal pH including fosinopril, mitotane, and mannitol.

Second, the LPLA_2_ inhibition assay identified several CADs known to cause phospholipidosis but that are not predicted to do so by use of in silico models based on the pKa and ClogP of CADs. The measurement of LPLA_2_ inhibition as a stand-alone assay for prediction of DIP is associated with a greater sensitivity and accuracy than models based on ClogP and pKa alone but slightly less than when these models are combined with an in vitro assay (28). Specifically, LPLA_2_ inhibition with an observed IC_50_ < 500 μM is 86% accurate in predicting phospholipidosis compared with the 58 and 79% accuracies of the Ploemen and modified Ploemen models, respectively. The accuracy in predicting phospholipidosis is greater than 90% for any observed inhibition of LPLA_2_ (Table 5). Individual drugs that cause phospholipidosis may do so synergistically (116), and such drugs may achieve concentrations within the lysosome that are up to 50,000-fold greater than that measured extracellularly (117). It is therefore possible that compounds that inhibit LPLA_2_ may do so at concentrations significantly greater than those associated with their therapeutic activity.

**Table 5:** LPLA_2_ assay sensitivity, specificity, and accuracy in comparison to those based on the Ploemen criteria

	Sensitivity/Specificity	PPV/NPV	False Positives	False Negatives	Accuracy
Ploemen model	55/87	96.3/24	atropine, 18 beta-glycyrrhetinic acid, S-methylisothiourea	azaperone, benzbromarone, bromocriptine, carbamazepine, clozapine, conessin, corticosterone, cyclopentolate, diclofenac, 5,7-dichloro-8-hydroxy-2-methyl quinolone, diphenhydramine, dutasteride, erythromycin, etomidate, fenofibrate, fipexide, flunarizine, flufenamic acid, fosinopril, fosinoprilat, fulvestrant, fusidic acid, gabapentin, gentisic acid, hydralazine, hydroxyzine, 6-hydroxydopamine, 3-hydroxytyramine, isoxsuprine, ketoconazole, ketotifen, lofepramine, loratadine, mannitol, mebhydrolin, melatonin, mianserin, mirtazapine, mitotane, oxolamine citrate, pantoprazole, penfluridol, perphenazine, phenacetin, phenytoin, pipamperone, pirenperone, pranlukast, progesterone, proparacaine, pyrilamine, repaglinide, ritanserin, spiperone, sulindac, sulpiride, suramin, tacrine, tetracaine, warfarin, yohimbine, zafirlukast	58.2
Modified Ploemen model	76.4/95.7	99.1/40	18 beta-glycyrrhetinic acid	benzbromarone, carbamazepine, conessin, corticosterone, diclofenac, 5,7 dichloro-8-hydroxy-2-methyl quinolone, etomidate, fenofibrate, fipexide, flufenamic acid, fosinopril, fosinoprilat, fulvestrant, fusidic acid, gabapentin, gentisic acid, hydralazine, 6-hydroxydopamine, 3-hydroxytyramine, loratadine, mannitol, melatonin, mitotane, pantoprazole, phenacetin, phenytoin, pranlukast, progesterone, sulindac, sulpiride, suramin, warfarin, zafirlukast	79.1
LPLA_2_ (IC_50_ ≤ 500 μM)	87.0/78.2	96/50	carbamazepine, imiquimod, ritanserin, rolipram, ropinirole	azaperone, betaxolol, chloroquine (655), clenbuterol (7298), clindamycin, 5,7 dichloro-8-hydroxy-2-methyl quinolone (1179), disopyramide, dutasteride (1048), etomidate (1155), isoxsuprine, lidocaine, methapyrilene, phenacetin (2140), pranlukast, procyclidine (1470), proparacaine (2469), sulpiride, vinblastine (2123)	85.9
LPLA_2_ (IC_50_ any concentration)	92.8/78.2	96.2/64	carbamazepine, imiquimod, ritanserin, rolipram, ropinirole	azaperone, betaxolol, clindamycin, disopyramide, isoxsuprine, lidocaine, methapyrilene, pranlukast, sulpiride	90.7

The table compares the sensitivity, specificity, positive predictive value (PPV), negative predictive value (NPV), and accuracy of the Ploemen, modified Ploemen, and LPLA2 assays. Compounds associated with false-positive and false-negative results are listed. The numbers in parentheses denote the μM IC50 for LPLA2 inhibition.

Third, LPLA_2_ inhibition may identify chemical entities currently approved by regulatory agencies that cause phospholipidosis but not previously identified as such. This is exemplified in the current study by the potent inhibition of LPLA_2_ by fosinopril and its active metabolite fosinoprilat and by validation of their phospholipidotic potential in the LipidTOX Red phospholipidosis assay. The IC_50_ value for fosinopril is significantly lower than that of the other drugs assayed in this study. Fosinopril is unique among the larger class of ACE inhibitors in that it contains a phosphinic-acid-containing ester that serves as the binding group as opposed to the more common carboxyl or sulfhydryl functions that characterize other ACE inhibitors (118). It is also among the most lipophilic of this class of drugs. Like amiodarone, the mechanism of inhibition by fosinopril appears to occur by interference of binding between LPLA_2_ and liposomes as supported by observed inhibition of cosedimentation of LPLA_2_ and liposomes in the presence of fosinopril. However, fosinopril is an amide and as a weak base not protonated at lysosomal pH unlike cationic amphiphilic drugs. Thus a different mechanism of inhibition that is distinct from amiodarone is likely in this case.

While there is general agreement that phospholipidosis results from the lysosomal accumulation of CADs, there is less agreement regarding the cause. LPLA_2_ is a good candidate for a cellular target by drugs that cause phospholipidosis. Although LPLA_2_ was first characterized as a phospholipase with transacylase activity toward short-chain ceramide acceptors (17), it was later recognized to be a phospholipase A2 with an acidic pH optimum (18). The further characterization of the enzymatic activity revealed broad substrate specificity to several glycerophospholipids including phosphatidylcholine, phosphatidylethanolamine, phosphatidylserine, and phosphatidylglycerol. Subsequent work characterized LPLA_2_ as having both PLA_1_ and PLA_2_ activity (119).

The earliest observed phenotype of a transgenic mouse knocked out for LPLA_2_ was the presence of alveolar macrophages with a foam cell appearance. Lipid analyses of both the macrophages and bronchoalveolar lavage fluid demonstrated increased levels of glycerophospholipids that were substrates for LPLA_2_ (20). The pulmonary toxicity associated with amiodarone is consistent with several of these functions. In its classic form, amiodarone toxicity is manifest as the accumulation of lipid-laden alveolar macrophages. The ultrastructure of these foam cells is characterized by the presence of lamellar bodies within lysosomes. Because the knockout mouse phenotype bore a strong resemblance to that seen with pulmonary amiodarone toxicity, the possible inhibition of LPLA_2_ by amiodarone was further studied (21). We observed at that time that amiodarone was not a direct inhibitor of LPLA_2_, but appeared to block the electrostatic interaction between liposomes and enzyme. This mechanism was further supported by the loss of activity in the presence of buffers of higher ionic content.

More recently, we determined a structure of LPLA_2_ by X-ray diffraction (22). The presence of the catalytic triad and the disulfide bond previously characterized was confirmed (120). Two tracks could accommodate the phospholipid head groups of a broad range of substrates. In the current model, *sn*-1 and *sn*-2 fatty acyl groups of these phospholipids can be oriented in track A within the catalytic domain and be recognized as the scissile fatty acyl group. This model is supported by the formation of 1-O-acyl-ceramides that are products of either *sn*-1 or *sn*-2 acyl groups on phospholipid substrates (121). The structural studies identified a distinct lipid binding domain and four cationic residues within the domain that are required for liposome binding. The observation that each of these residues was necessary for LPLA_2_ activity lent further support for the proposed mechanism of inhibition by amiodarone. Acid sphingomyelinase has also been identified as another target for DIP. A role for acid sphingomyelinase is also supported by the possibility that the substrate recognition of this phospholipase C may extend to phospholipids and beyond sphingomyelin. However, while the phospholipase C activity of acid sphingomyelinase may extend to phosphatidylcholine as well as sphingomyelin, the comparative activity is an order of magnitude greater for sphingomyelin (122, 123).

Although the inhibition of both LPLA_2_ and lysosomal acid sphingomyelinase by drugs that cause phospholipidosis has been proposed to occur by inhibition of electrostatic interactions between the respective phospholipases and anionic lipids, this model would not explain the phospholipidosis observed by compounds that are not basic drugs. This is exemplified in the present study by mitotane and mannitol, which lack amines and thus have no assignable pKa.

DIP has been an active focus of regulatory agencies including the FDA for more than 20 years. In 2004, the FDA announced that it formed an initiative named the Phospholipidosis Working Group under the auspices of the Center for Drug Evaluation and Research (124). The overarching goal of this group was to establish regulatory guidance for drugs that were observed to cause phospholipidosis. Significant research was fostered by this initiative leading to new in silico and in vitro tests, studies on the relation between CAD and DIP, efforts to understand potential connections between phospholipidosis and other toxicities such as QT prolongation and protein trafficking defects, and biomarker development including bis(monoacylglycerol) phosphate. However, these efforts did not provide a consensus as to a common mechanism or cellular target for CADs that cause phospholipidosis.

The identification here that LPLA_2_ inhibition is a primary basis for DIP provides further insight into the toxicological significance of DIP. While over 50 inherited monogenic lysosomal disorders have been identified, no clinical phenotype has yet to be described for an inherited loss of LPLA_2_ activity. However, a variety of potentially important biological roles for LPLA_2_ have been reported suggesting that long-term LPLA_2_ inhibition may be a pathological significance. These include a role for LPLA_2_ in surfactant degradation (20, 125), catabolism of oxidized phospholipids (126), ocular inflammation (127), host response to tuberculosis (128), and lipid antigen presentation through CD1d (129). While LPLA_2_ is expressed ubiquitously, the high activity of the phospholipase A2 in macrophages and other antigen presenting cells is consistent with an important role in host defense and antigen processing. Whether or not prolonged exposure to CADs that inhibit LPLA_2_ confers increased risk to loss of these functions will require further evaluation. Importantly, the recognition that LPLA_2_ is a primary target for DIP should aid in discerning drug-specific toxicities that are independent of LPLA_2_ inhibition and the result of other off-target effects.

Finally, the recognition that LPLA_2_ is the primary target for DIP raises the possibility that variants in the LPLA_2_ gene may account for differences in susceptibility to drugs that cause phospholipidosis within the population. Numerous sequence and splice variants have been identified for LPLA_2_, several of which are in the open reading frame of the LPLA_2_ gene and would predictably change the activity of the lipase either by resulting in the loss of catalytic activity or by conformational changes affecting the lipid-binding domain. Amiodarone, a highly effective antiarrhythmic, would be an obvious agent to study as DIP often limits its use. Future questions of interest might focus on establishing whether intrinsic differences in LPLA_2_ activity due to these variants account for susceptibility to amiodarone toxicity and whether structure activity studies of amiodarone might identify analogues that eliminate LPLA_2_ inhibition while maintaining antiarrhythmic activity.

## Data availability

All data are contained within the article and supplemental data.

## Supplemental data

This article contains supplemental data.

## Conflict of interest

The authors declare that they have no conflicts of interest with the contents of this article. Recombinant LPLA_2_ and anti-LPLA_2_ monoclonal antibodies are licensed to Echelon Biosciences by the University of Michigan.

## References

[1] Anderson N., Borlak J. (2006). Drug-induced phospholipidosis. FEBS Lett..

[2] Shayman J.A., Abe A. (2013). Drug induced phospholipidosis: an acquired lysosomal storage disorder. Biochim. Biophys. Acta.

[3] Chen G.L., Sutrina S.L., Frayer K.L., Chen W.W. (1986). Effects of lysosomotropic agents on lipogenesis. Arch. Biochem. Biophys..

[4] Kubo M., Hostetler K.Y. (1985). Mechanism of cationic amphiphilic drug inhibition of purified lysosomal phospholipase A1. Biochemistry.

[5] Ikeda K., Hirayama M., Hirota Y., Asa E., Seki J., Tanaka Y. (2008). Drug-induced phospholipidosis is caused by blockade of mannose 6-phosphate receptor-mediated targeting of lysosomal enzymes. Biochem. Biophys. Res. Commun..

[6] Hurwitz R., Ferlinz K., Sandhoff K. (1994). The tricyclic antidepressant desipramine causes proteolytic degradation of lysosomal sphingomyelinase in human fibroblasts. Biol. Chem. Hoppe Seyler.

[7] Joshi U.M., Kodavanti P.R., Coudert B., Dwyer T.M., Mehendale H.M. (1988). Types of interaction of amphiphilic drugs with phospholipid vesicles. J. Pharmacol. Exp. Ther..

[8] Henry B., Ziobro R., Becker K.A., Kolesnick R., Gulbins E. (2013). Acid sphingomyelinase. Handb. Exp. Pharmacol..

[9] Gonzalez A.C., Schweizer M., Jagdmann S., Bernreuther C., Reinheckel T., Saftig P., Damme M. (2018). Unconventional trafficking of mammalian phospholipase D3 to lysosomes. Cell Rep..

[10] Shayman J.A., Tesmer J.J.G. (2019). Lysosomal phospholipase A2. Biochim. Biophys. Acta Mol. Cell Biol. Lipids.

[11] Pelletier D.J., Gehlhaar D., Tilloy-Ellul A., Johnson T.O., Greene N. (2007). Evaluation of a published in silico model and construction of a novel Bayesian model for predicting phospholipidosis inducing potential. J. Chem. Inf. Model.

[12] Kruhlak N.L., Choi S.S., Contrera J.F., Weaver J.L., Willard J.M., Hastings K.L., Sancilio L.F. (2008). Development of a phospholipidosis database and predictive quantitative structure-activity relationship (QSAR) models. Toxicol. Mech. Methods.

[13] Orogo A.M., Choi S.S., Minnier B.L., Kruhlak N.L. (2012). Construction and consensus performance of (Q)sar models for predicting phospholipidosis using a dataset of 743 compounds. Mol. Inform.

[14] Choi S.S., Kim J.S., Valerio L.G., Sadrieh N. (2013). In silico modeling to predict drug-induced phospholipidosis. Toxicol. Appl. Pharmacol..

[15] Tengstrand E.A., Miwa G.T., Hsieh F.Y. (2010). Bis(monoacylglycerol)phosphate as a non-invasive biomarker to monitor the onset and time-course of phospholipidosis with drug-induced toxicities. Expert Opin. Drug Metab. Toxicol..

[16] Sawada H., Takami K., Asahi S. (2005). A toxicogenomic approach to drug-induced phospholipidosis: analysis of its induction mechanism and establishment of a novel in vitro screening system. Toxicol. Sci..

[17] Abe A., Shayman J.A., Radin N.S. (1996). A novel enzyme that catalyzes the esterification of N-acetylsphingosine. Metabolism of C2-ceramides. J. Biol. Chem..

[18] Abe A., Shayman J.A. (1998). Purification and characterization of 1-O-acylceramide synthase, a novel phospholipase A2 with transacylase activity. J. Biol. Chem..

[19] Hiraoka M., Abe A., Shayman J.A. (2002). Cloning and characterization of a lysosomal phospholipase A2, 1-O-acylceramide synthase. J. Biol. Chem..

[20] Hiraoka M., Abe A., Lu Y., Yang K., Han X., Gross R.W., Shayman (2006). Lysosomal phospholipase A2 and phospholipidosis. Mol. Cell Biol..

[21] Abe A., Hiraoka M., Shayman J.A. (2007). A role for lysosomal phospholipase A2 in drug induced phospholipidosis. Drug Metab. Lett..

[22] Glukhova A., Hinkovska-Galcheva V., Kelly R., Abe A., Shayman J.A., Tesmer J.J. (2015). Structure and function of lysosomal phospholipase A2 and lecithin:cholesterol acyltransferase. Nat. Commun..

[23] Shayman J.A., Abe A. (2000). 1-O-acylceramide synthase. Methods Enzymol..

[24] Hinkovska-Galcheva V., Kelly R., Manthei K.A., Bouley R., Yuan W., Schwendeman A., Tesmer J.J.G., Shayman1 J.A. (2018). Determinants of pH profile and acyl chain selectivity in lysosomal phospholipase A2. J. Lipid Res..

[25] Abe A., Shayman J.A. (2009). The role of negatively charged lipids in lysosomal phospholipase A2 function. J. Lipid Res..

[26] Semisotnov G.V., Rodionova N.A., Razgulyaev O.I., Uversky V.N., Gripas A.F., Gilmanshin R.I. (1991). Study of the "molten globule" intermediate state in protein folding by a hydrophobic fluorescent probe. Biopolymers.

[27] Nioi P., Perry B.K., Wang E.J., Gu Y.Z., Snyder R.D. (2007). In vitro detection of drug-induced phospholipidosis using gene expression and fluorescent phospholipid based methodologies. Toxicol. Sci..

[28] Muehlbacher M., Tripal P., Roas F., Kornhuber J. (2012). Identification of drugs inducing phospholipidosis by novel in vitro data. ChemMedChem.

[29] Treyer A., Mateus A., Wisniewski J.R., Boriss H., Matsson P., Artursson P. (2018). Intracellular drug bioavailability: effect of neutral lipids and phospholipids. Mol. Pharm..

[30] Kornhuber J., Muehlbacher M., Trapp S., Pechmann S., Friedl A., Reichel M., Mühle C., Terfloth L., Groemer T.W., Spitzer G.M., Liedl K.R., Gulbins E., Tripal P. (2011). Identification of novel functional inhibitors of acid sphingomyelinase. PLoS One.

[31] Heath M.F., Jacobson W. (1985). The inhibition of lysosomal phospholipase A from rabbit lung by ambroxol and its consequences for pulmonary surfactant. Lung.

[32] Lenhard S.C., Lev M., Webster L.O., Peterson R.A., Goulbourne C.N., Miller R.T., Jucker B.M. (2016). Hepatic phospholipidosis is associated with altered hepatobiliary function as assessed by gadoxetate dynamic contrast-enhanced magnetic resonance imaging. Toxicol. Pathol..

[33] Gonzalez-Rothi R.J., Hannan S.E., Hood C.I., Franzini D.A. (1987). Amiodarone pulmonary toxicity presenting as bilateral exudative pleural effusions. Chest.

[34] Fischer H., Atzpodien E.A., Csato M., Doessegger L., Lenz B., Schmitt G., Singer T. (2012). In silico assay for assessing phospholipidosis potential of small druglike molecules: training, validation, and refinement using several data sets. J. Med. Chem..

[35] Morelli J.K., Buehrle M., Pognan F., Barone L.R., Fieles W., Ciaccio P.J. (2006). Validation of an in vitro screen for phospholipidosis using a high-content biology platform. Cell Biol. Toxicol..

[36] Hanumegowda U.M., Wenke G., Regueiro-Ren A., Yordanova R., Corradi J.P., Adams S.P. (2010). Phospholipidosis as a function of basicity, lipophilicity, and volume of distribution of compounds. Chem. Res. Toxicol..

[37] Shahane S.A., Huang R., Gerhold D., Baxa U., Austin C.P., Xia M. (2014). Detection of phospholipidosis induction: a cell-based assay in high-throughput and high-content format. J. Biomol. Screen.

[38] Tochitani T., Yamashita A., Kouchi M., Fujii Y., Matsumoto I., Miyawaki I., Yamada T., Funabashi H. (2016). Changes in plasma concentrations of corticosterone and its precursors after ketoconazole administration in rats: An application of simultaneous measurement of multiple steroids using LC-MS/MS. Exp. Toxicol. Pathol..

[39] Atienzar F., Gerets H., Dufrane S., Tilmant K., Cornet M., Dhalluin S., Ruty B., Rose G., Canning M. (2007). Determination of phospholipidosis potential based on gene expression analysis in HepG2 cells. Toxicol. Sci..

[40] Przybylak K.R., Cronin M.T. (2011). In silico studies of the relationship between chemical structure and drug induced phospholipidosis. Mol. Inform.

[41] Zapata E., Zubiaurre L., Bujanda L., Pierola A. (2006). Anastrozole-induced hepatotoxicity. Eur. J. Gastroenterol. Hepatol..

[42] Hozumi Y., Suemasu K., Takei H., Aihara T., Takehara M., Saito T., Ohsumi S., Masuda N., Ohashi Y. (2011). The effect of exemestane, anastrozole, and tamoxifen on lipid profiles in Japanese postmenopausal early breast cancer patients: final results of National Surgical Adjuvant Study BC 04, the TEAM Japan sub-study. Ann. Oncol..

[43] Yoshida Y., Arimoto K., Sato M., Sakuragawa N., Arima M., Satoyoshi E. (1985). Reduction of acid sphingomyelinase activity in human fibroblasts induced by AY-9944 and other cationic amphiphilic drugs. J. Biochem..

[44] Halliwell W.H. (1997). Cationic amphiphilic drug-induced phospholipidosis. Toxicol. Pathol..

[45] Kikkawa Y., Motoyama E.K. (1973). Effect of AY-9944, a cholesterol biosynthesis inhibitor, on fetal lung development and on the development of type II alveolar epithelial cells. Lab. Invest..

[46] Rawlins F.A., Uzman B.G. (1970). Effect of AY-9944, a cholesterol biosynthesis inhibitor, on peripheral nerve myelination. Lab. Invest..

[47] Frachon I., Le Gal G., Hill C., Leroyer C. (2011). Benfluorex withdrawal in France: still be hiding somewhere in the world?. J. Pharmacol. Pharmacother..

[48] Kohl C., Ravel D., Girard J., Pegorier J.P. (2002). Effects of benfluorex on fatty acid and glucose metabolism in isolated rat hepatocytes: from metabolic fluxes to gene expression. Diabetes.

[49] Fusani L., Brown M., Chen H., Ahlberg E., Noeske T. (2017). Predicting the risk of phospholipidosis with in silico models and an image-based in vitro screen. Mol. Pharm..

[50] Eckert H., Lux M., Lachmann B. (1983). The role of alveolar macrophages in surfactant turnover. An experimental study with metabolite VIII of bromhexine (Ambroxol). Lung.

[51] Frohlich E. (2017). Toxicity of orally inhaled drug formulations at the alveolar barrier: parameters for initial biological screening. Drug Deliv..

[52] Pappu A.S., Yazaki P.J., Hostetler K.Y. (1985). Inhibition of purified lysosomal phospholipase A1 by beta-adrenoceptor blockers. Biochem. Pharmacol..

[53] Gregory M.H., Rutty D.A., Wood R.D. (1970). Differences in the retinotoxic action of chloroquine and phenothiazine derivatives. J. Pathol..

[54] Rosenthal A.R., Kolb H., Bergsma D., Huxsoll D., Hopkins J.L. (1978). Chloroquine retinopathy in the rhesus monkey. Invest. Ophthalmol. Vis. Sci..

[55] Meanwell N.A. (2018). Fluorine and fluorinated motifs in the design and application of bioisosteres for drug design. J. Med. Chem..

[56] Low Y., Uehara T., Minowa Y., Yamada H., Ohno Y., Urushidani T., Sedykh A., Muratov E., Kuz'min V., Fourches D., Zhu H., Rusyn I., Tropsha A. (2011). Predicting drug-induced hepatotoxicity using QSAR and toxicogenomics approaches. Chem. Res. Toxicol..

[57] Kornhuber J., Henkel A.W., Groemer T.W., Stadtler S., Welzel O., Tripal P., Rotter A., Bleich S., Trapp S. (2010). Lipophilic cationic drugs increase the permeability of lysosomal membranes in a cell culture system. J. Cell Physiol..

[58] Baik J., Rosania G.R. (2012). Macrophages sequester clofazimine in an intracellular liquid crystal-like supramolecular organization. PLoS One.

[59] Baik J., Stringer K.A., Mane G., Rosania G.R. (2013). Multiscale distribution and bioaccumulation analysis of clofazimine reveals a massive immune system-mediated xenobiotic sequestration response. Antimicrob. Agents Chemother..

[60] Xu L., Sheflin L.G., Porter N.A., Fliesler S.J. (2012). 7-Dehydrocholesterol-derived oxysterols and retinal degeneration in a rat model of Smith-Lemli-Opitz syndrome. Biochim. Biophys. Acta.

[61] Talamo J.H., D'Amico D.J., Hanninen L.A., Kenyon K.R., Shanks E.T. (1985). The influence of aphakia and vitrectomy on experimental retinal toxicity of aminoglycoside antibiotics. Am. J. Ophthalmol..

[62] Scuntaro I., Kientsch U., Wiesmann U.N., Honegger U.E. (1996). Inhibition by vitamin E of drug accumulation and of phospholipidosis induced by desipramine and other cationic amphiphilic drugs in human cultured cells. Br. J. Pharmacol..

[63] Kornhuber J., Tripal P., Gulbins E., Muehlbacher M. (2013). Functional inhibitors of acid sphingomyelinase (FIASMAs). Handb. Exp. Pharmacol..

[64] MacNeil D.J. (1997). The side effect profile of class III antiarrhythmic drugs: focus on d,l-sotalol. Am. J. Cardiol..

[65] Gray J.E., Purmalis A., Purmalis B., Mathews J. (1971). Ultrastructural studies of the hepatic changes brought about by Clindamycin and Erythromycin in animals. Toxicol. Appl. Pharmacol..

[66] Qi C., Zhu Y., Reddy J.K. (2000). Peroxisome proliferator-activated receptors, coactivators, and downstream targets. Cell Biochem. Biophys..

[67] Perez Martin J.M., Labrador V., Fernandez Freire P., Molero M.L., Hazen M.J. (2004). Ultrastructural changes induced in HeLa cells after phototoxic treatment with harmine. J. Appl. Toxicol..

[68] Chan K., Truong D., Shangari N., O'Brien P.J. (2005). Drug-induced mitochondrial toxicity. Expert Opin. Drug Metab. Toxicol..

[69] Kazmi F., Hensley T., Pope C., Funk R.S., Loewen G.J., Buckley D.B., Parkinson A. (2013). Lysosomal sequestration (trapping) of lipophilic amine (cationic amphiphilic) drugs in immortalized human hepatocytes (Fa2N-4 cells). Drug Metab. Dispos.

[70] Bendele R.A., Adams E.R., Hoffman W.P., Gries C.L., Morton D.M. (1992). Carcinogenicity studies of fluoxetine hydrochloride in rats and mice. Cancer Res..

[71] McMillian M.K., Grant E.R., Zhong Z., Parker J.B., Li L., Zivin R.A., Burczynski M.E., Johnson M.D. (2001). Nile Red binding to HepG2 cells: an improved assay for in vitro studies of hepatosteatosis. In Vitr. Mol. Toxicol..

[72] Ginoulhiac E., Semenza F., Mainardi L. (1950). [Toxicity and pharmacologic effects of gentisic acid]. Boll Soc. Ital. Biol. Sper.

[73] Hruban Z., Slesers A., Hopkins E. (1972). Drug-induced and naturally occurring myeloid bodies. Lab. Invest..

[74] Drenckhahn D., Lullmann-Rauch R. (1979). Experimental myopathy induced by amphiphilic cationic compounds including several psychotropic drugs. Neuroscience.

[75] Glassman A.H., Perel J.M. (1973). The clinical pharmacology of imipramine. Implications for therapeutics. Arch. Gen. Psychiatry.

[76] Reasor M.J. (1989). A review of the biology and toxicologic implications of the induction of lysosomal lamellar bodies by drugs. Toxicol. Appl. Pharmacol..

[77] Salabei J.K., Balakumaran A., Frey J.C., Boor P.J., Treinen-Moslen M., Conklin D.J. (2012). Verapamil stereoisomers induce antiproliferative effects in vascular smooth muscle cells via autophagy. Toxicol. Appl. Pharmacol..

[78] Takagi M., Sanoh S., Santoh M., Ejiri Y., Kotake Y., Ohta S. (2016). Detection of metabolic activation leading to drug-induced phospholipidosis in rat hepatocyte spheroids. J. Toxicol. Sci..

[79] Zheng N., Zhang X., Rosania G.R. (2011). Effect of phospholipidosis on the cellular pharmacokinetics of chloroquine. J. Pharmacol. Exp. Ther..

[80] Staubli W., Schweizer W., Suter J., Hess R. (1974). Ultrastructural and biochemical study of the action of benzoctamine and maprotiline on the rat liver. Agents Actions.

[81] Park S., Choi Y.J., Lee B.H. (2012). In vitro validation of drug-induced phospholipidosis. J. Toxicol. Sci..

[82] Honegger U.E., Quack G., Wiesmann U.N. (1993). Evidence for lysosomotropism of memantine in cultured human cells: cellular kinetics and effects of memantine on phospholipid content and composition, membrane fluidity and beta-adrenergic transmission. Pharmacol. Toxicol..

[83] Telles-Correia D., Barbosa A., Cortez-Pinto H., Campos C., Rocha N.B., Machado S. (2017). Psychotropic drugs and liver disease: a critical review of pharmacokinetics and liver toxicity. World J. Gastrointest. Pharmacol. Ther..

[84] Murakami M., Sato H., Miki Y., Yamamoto K., Taketomi Y. (2015). A new era of secreted phospholipase A(2). J. Lipid Res..

[85] Waszut U., Szyszka P., Dworakowska D. (2017). Understanding mitotane mode of action. J. Physiol. Pharmacol..

[86] Kirilmaz L., Kendirci A., Guneri T. (1992). Sustained-release dosage form of oxolamine citrate: preparation and release kinetics. J. Microencapsul.

[87] Pintavorn P., Cook W.J. (2008). Progressive renal insufficiency associated with amiodarone-induced phospholipidosis. Kidney Int..

[88] Fardeau M., Tome F.M., Simon P. (1979). Muscle and nerve changes induced by perhexiline maleate in man and mice. Muscle Nerve.

[89] Lullmann H., Lullmann-Rauch R. (1978). Perhexiline induces generalized lipidosis in rats. Klin. Wochenschr..

[90] Lullmann H., Lullmann-Rauch R., Wassermann O. (1978). Lipidosis induced by amphiphilic cationic drugs. Biochem. Pharmacol..

[91] Pakuts A.P., Parks R.J., Paul C.J., Bujaki S.J., Mueller R.W. (1990). Ketoconazole-induced hepatic lysosomal phospholipidosis: the effect of concurrent barbiturate treatment. Res. Commun. Chem. Pathol. Pharmacol..

[92] Gonzalez-Rothi R.J., Zander D.S., Ros P.R. (1995). Fluoxetine hydrochloride (Prozac)-induced pulmonary disease. Chest.

[93] Schlecht U., St Onge R.P., Walther T., Francois J.M., Davis R.W. (2012). Cationic amphiphilic drugs are potent inhibitors of yeast sporulation. PLoS One.

[94] Chapy H., Goracci L., Vayer P., Parmentier Y., Carrupt P.A., Decleves X., Scherrmann J.M., Cisternino S., Cruciani G. (2015). Pharmacophore-based discovery of inhibitors of a novel drug/proton antiporter in human brain endothelial hCMEC/D3 cell line. Br. J. Pharmacol..

[95] Folts C.J., Scott-Hewitt N., Proschel C., Mayer-Proschel M., Noble M. (2016). Lysosomal re-acidification prevents lysosphingolipid-induced lysosomal impairment and cellular toxicity. PLoS Biol..

[96] Vejux A., Malvitte L., Lizard G. (2008). Side effects of oxysterols: cytotoxicity, oxidation, inflammation, and phospholipidosis. Braz. J. Med. Biol. Res..

[97] Slavov S.H., Wilkes J.G., Buzatu D.A., Kruhlak N.L., Willard J.M., Hanig J.P., Beger R.D. (2014). Computational identification of a phospholipidosis toxicophore using (13)C and (15)N NMR-distance based fingerprints. Bioorg. Med. Chem..

[98] Hruban Z. (1984). Pulmonary and generalized lysosomal storage induced by amphiphilic drugs. Environ. Health Perspect..

[99] Zidovetzki R., Sherman I.W., Atiya A., De Boeck H. (1989). A nuclear magnetic resonance study of the interactions of the antimalarials chloroquine, quinacrine, quinine and mefloquine with dipalmitoylphosphatidylcholine bilayers. Mol. Biochem. Parasitol..

[100] Song M., Kim Y.J., Ryu J.C. (2011). Phospholipidosis induced by PPARgamma signaling in human bronchial epithelial (BEAS-2B) cells exposed to amiodarone. Toxicol. Sci..

[101] Rayburn E.R., Gao L., Ding J., Ding H., Shao J., Li H. (2018). FDA-approved drugs that are spermatotoxic in animals and the utility of animal testing for human risk prediction. J. Assist Reprod. Genet..

[102] Sahini N., Selvaraj S., Borlak J. (2014). Whole genome transcript profiling of drug induced steatosis in rats reveals a gene signature predictive of outcome. PLoS One.

[103] Wang T., Feng Y., Jin X., Fan X., Crommen J., Jiang Z. (2014). Liposome electrokinetic chromatography based in vitro model for early screening of the drug-induced phospholipidosis risk. J. Pharm. Biomed. Anal.

[104] Soldani P., Pellegrini A., Gesi M., Lenzi P., Paparelli A. (1996). Suramin-induced ultrastructural changes in the testis of albino rats. Exp. Toxicol. Pathol..

[105] Raizman M.B., Hamrah P., Holland E.J., Kim T., Mah F.S., Rapuano C.J., Ulrich R.G. (2017). Drug-induced corneal epithelial changes. Surv. Ophthalmol..

[106] Bhandari N., Figueroa D.J., Lawrence J.W., Gerhold D.L. (2008). Phospholipidosis assay in HepG2 cells and rat or rhesus hepatocytes using phospholipid probe NBD-PE. Assay Drug Dev. Technol..

[107] Goracci L., Ceccarelli M., Bonelli D., Cruciani G. (2013). Modeling phospholipidosis induction: reliability and warnings. J. Chem. Inf. Model.

[108] De Broe M.E., Paulus G.J., Verpooten G.A., Roels F., Buyssens N., Wedeen R., Van Hoof F., Tulkens P.M. (1984). Early effects of gentamicin, tobramycin, and amikacin on the human kidney. Kidney Int..

[109] Toubeau G., Maldague P., Laurent G., Vaamonde C.A., Tulkens P.M., Heuson-Stiennon J.A. (1986). Morphological alterations in distal and collecting tubules of the rat renal cortex after aminoglycoside administration at low doses. Virchows Arch. B Cell Pathol. Incl. Mol. Pathol..

[110] Yates R.D., Arai K., Rappoport D.A. (1967). Fine structure and chemical composition of opaque cytoplasmic bodies of triparanol treated Syrian hamsters. Exp. Cell Res..

[111] Breiden B., Sandhoff K. (2019). Emerging mechanisms of drug-induced phospholipidosis. Biol. Chem..

[112] Chen I.L., Yates R.D. (1967). An ultrastructural study of opague cytoplasmic inclusions induced by triparanol treatment. Am. J. Anat..

[113] Dietert S.E., Scallen T.J. (1969). An ultrastructural and biochemical study of the effects of three inhibitors of cholesterol biosynthesis upon murine adrenal gland and testis. Histochemical evidence for a lysosome response. J. Cell Biol..

[114] Tomizawa K., Sugano K., Yamada H., Horii I. (2006). Physicochemical and cell-based approach for early screening of phospholipidosis-inducing potential. J. Toxicol. Sci..

[115] Ploemen J.P., Kelder J., Hafmans T., van de Sandt H., van Burgsteden J.A., Saleminki P.J., van Esch E. (2004). Use of physicochemical calculation of pKa and CLogP to predict phospholipidosis-inducing potential: a case study with structurally related piperazines. Exp. Toxicol. Pathol..

[116] Glock M., Muehlbacher M., Hurtig H., Tripal P., Kornhuber J. (2016). Drug-induced phospholipidosis caused by combinations of common drugs in vitro. Toxicol. Vitro.

[117] Derendorf H. (2020). Excessive lysosomal ion-trapping of hydroxychloroquine and azithromycin. Int. J. Antimicrob. Agents.

[118] Piepho R.W. (2000). Overview of the angiotensin-converting-enzyme inhibitors. Am. J. Health Syst. Pharm..

[119] Abe A., Hiraoka M., Shayman J.A. (2006). Positional specificity of lysosomal phospholipase A2. J. Lipid Res..

[120] Hiraoka M., Abe A., Shayman J.A. (2005). Structure and function of lysosomal phospholipase A2: identification of the catalytic triad and the role of cysteine residues. J. Lipid Res..

[121] Hinkovska-Galcheva V., Kelly R., Manthei K.A., Bouley R., Yuan W., Schwendeman A., Tesmer J.J.G., Shayman J.A. (2018). Determinants of pH profile and acyl chain selectivity in lysosomal phospholipase A2. J. Lipid Res..

[122] Quintern L.E., Weitz G., Nehrkorn H., Tager J.M., Schram A.W., Sandhoff K. (1987). Acid sphingomyelinase from human urine: purification and characterization. Biochim. Biophys. Acta.

[123] Oninla V.O., Breiden B., Babalola J.O., Sandhoff K. (2014). Acid sphingomyelinase activity is regulated by membrane lipids and facilitates cholesterol transfer by NPC2. J. Lipid Res..

[124] Berridge B.R., Chatman L.A., Odin M., Schultze A.E., Losco P.E., Meehan J.T., Peters T., Vonderfecht S.L., Society of Toxicologic Pathology Scientific and Regulatory Policy Committee Working Group (2007). Phospholipidosis in nonclinical toxicity studies. Toxicol. Pathol..

[125] Abe A., Kelly R., Kollmeyer J., Hiraoka M., Lu Y., Shayman J.A. (2008). The secretion and uptake of lysosomal phospholipase A2 by alveolar macrophages. J. Immunol..

[126] Abe A., Hiraoka M., Ohguro H., Tesmer J.J., Shayman J.A. (2017). Preferential hydrolysis of truncated oxidized glycerophospholipids by lysosomal phospholipase A2. J. Lipid Res..

[127] Sawada K., Hiraoka M., Abe A., Kelly R., Shayman J.A., Ohguro H. (2017). Prolonged ocular inflammation in endotoxin-induced uveitis in lysosomal phospholipase A2-deficient mice. Curr. Eye Res..

[128] Schneider B.E., Behrends J., Hagens K., Harmel N., Shayman J.A., Schaible U.E. (2014). Lysosomal phospholipase A2: a novel player in host immunity to Mycobacterium tuberculosis. Eur. J. Immunol..

[129] Paduraru C., Bezbradica J.S., Kunte A., Kelly R., Shayman J.A., Veerapen N., Cox L.R., Besra G.S., Cresswell P. (2013). Role for lysosomal phospholipase A2 in iNKT cell-mediated CD1d recognition. Proc. Natl. Acad. Sci. U.S.A..

