# Supplementary material for: Inhibition of lysosomal phospholipase A2 predicts drug-induced phospholipidosis
Source: J Lipid Res. 2021 Jun 1;62:100089. doi: 10.1016/j.jlr.2021.100089 (PMC8243516; doi:10.1016/j.jlr.2021.100089)
Supplement: Supplemental Figures S1 and S2 and Supplemental Table S1 [file mmc1.docx]

Supporting information for Lysosomal phospholipase A2 is a primary target for drug induced phospholipidosis

Contents

Supplemental figure 1.

Supplemental figure 2.

Supplemental table 1.

**Supplemental figure 1. Fosinopril induces phospholipidosis in MDCK cells.** MDCK cells were co-treated with the indicated concentrations of fosinopril or amiodarone and LipidTOX Red for 24h prior to image acquisition. Compared to non-treated control (A), fosinopril (B) induced a significant increase in LipidTOX Red staining similar to that observed with the phospholipidosis-positive control, amiodarone (C). (D-F) Merged images revealing nuclei staining. Scale bar = 200μM.

**Supplemental figure 2. Quantification of fosinopril-induced phospholipidosis in MDCK cells.** Images were subjected to quantification, which revealed a significant increase in LipidTOX Red particle number (A) and mean fluorescent intensity (MFI) (B) in fosinopril- and amiodarone-treated conditions compared to non-treated control. No change in cell number was observed (C). **** denotes p< 0.0001.

**Supplemental Table 1. LPLA2 Thermal Shift**

| Drug | (ΔTm)_c_-(ΔTm)_d_^0^C | Drug | (ΔTm)_c_-(ΔTm)_d_^0^C |
| --- | --- | --- | --- |
| amisulpride | 0 | imiquimod | 0.66 ± 0.2 |
| allopurinol | 0.67 ± 0.2 | indoramin | 1.08 ± 0.4 |
| alprenolol | -1.00 ± 0.1 | isoxsuprine | 1.1 ± 0.0 |
| alverine | 0.14 ± 0.1 | ketoconazole | 0.42 ± 0.8 |
| ambroxol | 0.68 ± 0.5 | ketotifen | 0.10 ± 0.0 |
| amiodarone | -1.6 ± 0.1 | lercanidipine | 0.21 ± 0.3 |
| amitriptyline | -0.5 ± 0.0 | lidocaine | 1.59 ± 0.4 |
|  |  | l-leucine | 1.86 ± 0.6 |
| amorolfine | -0.30 ± 0.1 | lofepramine | 0 |
| anastrozole | -1.05 ± 0.1 | loperamide | 1.08 ± 0.1 |
| astemizole | -2.40 ± 0.4 | loratadine | -1.0 ± 0.1 |
| atovaquone | -0.17 ± 0.1 | mannitol | 1.0 ± 0.0 |
| atropine | 0.52 ± 0.0 | maprotiline | -0.5 ± 0.0 |
| ay-9944 | 0.58 ± 0.4 | mebeverine | -0.05 ± 0.5 |
| azaperone | 0.93 ± 0.1 | mebhydroline | 0.84 ± 0.1 |
| benzbromarone | -1.40 ± 0.3 | meclofenamic acid | -0.23 ± 0.1 |
| benfluorex | -2.40 ± 0.2 | memantine | 2.00 ± 0.2 |
| benztropine | 0.30 ± 0.1 | melatonin | 0.15 ± 0.1 |
| bepridil | 2.10 ± 0.6 | methapyrilene | -0.48 ± 0.2 |
| betaxolol | -1.29 ± 0.9 | mianserin | 1.00 ± 1.0 |
| 18 beta-glycyrrhetinic acid | 1.88 ± 0.6 | mibefradil | 1.30 ± 0.1 |
| bromhexine | 1.26 ± 0.2 | mifepristone | 0.20 ± 0.0 |
| bromocriptine | 2.39 ± 1.4 | mirtazapine | -1.30 ± 0.7 |
| butenafine | -2.22 ± 0.3 | mitotane | 0.47 ± 0.1 |
| buclizine | 0.63 ± 0.1 | naphazoline | 0 |
| carbamazepine | -1.26 ± 0.1 | naproxen | -0.19 ± 0.0 |
| chloroquine | 0.60 ± 0.0 | orphenadrine | -0.38 ± 0.3 |
| chlorpheniramine | 0.34 ± 0.0 | oxolamine | -0.89 ± 0.0 |
| chlorprothixene | -2.0 ± 0.1 | oxybutynin | 0.41 ± 0.0 |
| chlorpromazine | -2.1 ± 0.2 | pantoprazole | 0.25 ± 0.1 |
| cinnarizine | 0.54 ± 0.9 | paroxetine | 1.12 ± 0.1 |
| citalopram | 0 | penfluridol | 0.11 ± 0.0 |
| clemastine | 0.16 ± 0.9 | perhexiline | -0.12 ± 0.0 |
| clenbuterol | 1.20 ± 0.23 | perphenazine | -0.41 ± 0.2 |
| clindamycin | 0.95 ± 0.2 | phenacetin | -2.30 ± 0.6 |
| clofazimine | -0.97 ± 0.1 | pimozide | 0.26 ± 0.0 |
| clomifene | 1.07 ± 0.1 | phenytoin | 0.13 ± 0.1 |
| clomipramine | -0.30 ± 0.1 | pipamperone | 0.83 ± 0.1 |
| clonidine | -0.62 ± 0.0 | pirenperone | 0.79 ± 0.0 |
| cloperastin | 0 | pipamperone | 0.83 ± 0.1 |
| cloricromen | 0.24 ± 0.1 | pirenperone | 0.79 ± 0.1 |
| clozapine | -0.83 ± 0.0 | PP 06424439 | 0.80 ± 0.3 |
| conessin | 0.20 ± 01 | pranlukast | 0 |
| corticosterone | 0 | pridinol | 0 |
| cyclazosin | 0.18 ± 0.1 | prochlorperazine | -0.14 ± 0.1 |
| cyclobenzaprine | 2.50 ± 0.1 | procyclidine | 0.51 ± 0.0 |
| cyclopentolate | 0.87 ± 0.6 | profenamine | 2.40 ± 0.4 |
| desipramine | 1.60 ± 0.1 | progesterone | 0.61 ± 0.1 |
| desloratadine | 0.30 ± 0.1 | promazine | -0.10 ± 0.0 |
| dibenzosuberane | -1.94 ± 0.0 | promethazine | 1.45 ± 0.5 |
| diclofenac | 1.70 ± 0.5 | propafenone | -0.90 ± 0.1 |
| 5,7 dichloro-8-hydroxy-2-methyl quinolone | 1.88 ± 0.9 | proparacaine | 0 |
| dilazep | 0.11 ± 0.1 | propranolol | 1.03 ± 0.2 |
| 1,7-dimethylxanthine | 0 | pyrilamine | 0 |
| diphenhydramine | 0.70 ± 0.0 | quinacrine | 1.80 ± 0.3 |
| disopyramide | 0 | quinine | -1.00 ± 0.1 |
| doxepin | 0.12 ± 0.5 | repaglinide | -0.84 ± 1.5 |
| drofenine | 1.50 ± 0.1 | retinol | 0.81 ± 0.3 |
| dutasteride | -0.45 ± 0.0 | ritanserin | -1.90 ± 02 |
| encainide | -0.76 ± 0.1 | rolipram | -1.85 ± 0.9 |
| erythromycin | -0.48 ± 0.4 | ropinirole | 0 |
| etomidate | -0.76 ± 0.3 | sb222200 | -1.20 ± 0.1 |
| fenofibrate | 0.44 ± 0.7 | sertraline | -1.00 ± 03 |
| fenspiride | -1.67 ± 1.0 | s-methylisothiourea | -2.05 ± 0.6 |
| fexofenadine | -0.55 ± 0.0 | spiperone | 1.86 ± 0.1 |
| fipexide | -0.17 ± 0.0 | sulindac | -0.10 ± 0.0 |
| flunarizine | 1.30 ± 0.2 | suloctidil | 2.00 ± 0.2 |
| fluoxetine | -0.30 ± 0.1 | **sulpiride** | 1.34 ± 0.9 |
| flufenamic acid | -0.62 ± 0.3 | suramin | 0 |
| fosinopril | -0.90 ± 0.1 | tamoxifen | 0 |
| fosinoprilat | -1.45 ± 0.9 | tacrine | 0 |
| fulvestrant | -1.05 ± 4.0 | tetracaine | -1.20 ± 0.5 |
| fusidic acid | 0.94 ± 0.1 | thioridazine | -0.33 ± 0.1 |
| gabapentin | 0 | tobramycin | 0.27 ± 0.1 |
| gentisic acid | 1.33 ± 0.1 | trifluoperazine | 0.49 ± 0.3 |
| D-( ± )-glucose | 0 | trimipramine | 0.56 ± 0.0 |
| harmine | -1.94 ± 0.9 | triparanol | 0 |
| hydralazine | 1.20 ± 0.1 | uridine | -1.71 ± 0.1 |
| hydrocortisone | 1.11 ± 0.1 | vinblastine | -0.24 ± 0.0 |
| hydroxyzine | -0.10 ± 0.0 | warfarin | -0.65 ± 0.2 |
| 6-hydroxydopamine | -0.14 ± 0.1 | xylometazoline | 0.66 ± 0.0 |
| 3-hydroxytyramine | 0.75 ± 0.0 | yohimbine | 1.52 ± 0.6 |
| imipramine | 0 | zafirlukast | 1.80 ± 0.4 |

(ΔTm)_c_-(ΔTm)_d_^0^C denotes the difference in melting temperature of LPLA2 alone or in the presence of drug. N=3 ± SD.
